# Use of Phenomics for Differentiation of Mungbean (*Vigna radiata* L. Wilczek) Genotypes Varying in Growth Rates Per Unit of Water

**DOI:** 10.3389/fpls.2021.692564

**Published:** 2021-06-21

**Authors:** Jagadish Rane, Susheel Kumar Raina, Venkadasamy Govindasamy, Hanumantharao Bindumadhava, Prashantkumar Hanjagi, Rajkumar Giri, Krishna Kumar Jangid, Mahesh Kumar, Ramakrishnan M. Nair

**Affiliations:** ^1^School of Water Stress Management, Indian Council of Agricultural Research-National Institute of Abiotic Stress Management, Baramati, India; ^2^Indian Council of Agricultural Research-National Bureau of Plant Genetic Resources, Regional Station, Srinagar, India; ^3^Division of Microbiology, Indian Council of Agricultural Research-Indian Agricultural Research Institute, New Delhi, India; ^4^World Vegetable Center, South Asia, International Crops Research Institute for the Semi-Arid Tropics Campus, Hyderabad, India; ^5^Marri Channa Reddy Foundation (MCRF), Hyderabad, India; ^6^Division of Crop Physiology and Biochemistry, Indian Council of Agricultural Research-National Rice Research Institute, Cuttack, India

**Keywords:** high throughput phenotyping, plant phenomics, growth rate, mungbean [*Vigna radiata* (L.) Wilczek], drought, water use index, soil moisture stress

## Abstract

In the human diet, particularly for most of the vegetarian population, mungbean (*Vigna radiata* L. Wilczek) is an inexpensive and environmentally friendly source of protein. Being a short-duration crop, mungbean fits well into different cropping systems dominated by staple food crops such as rice and wheat. Hence, knowing the growth and production pattern of this important legume under various soil moisture conditions gains paramount significance. Toward that end, 24 elite mungbean genotypes were grown with and without water stress for 25 days in a controlled environment. Top view and side view (two) images of all genotypes captured by a high-resolution camera installed in the high-throughput phenomics were analyzed to extract the pertinent parameters associated with plant features. We tested eight different multivariate models employing machine learning algorithms to predict fresh biomass from different features extracted from the images of diverse genotypes in the presence and absence of soil moisture stress. Based on the mean absolute error (MAE), root mean square error (RMSE), and R squared (*R*^2^) values, which are used to assess the precision of a model, the partial least square (PLS) method among the eight models was selected for the prediction of biomass. The predicted biomass was used to compute the plant growth rates and water-use indices, which were found to be highly promising surrogate traits as they could differentiate the response of genotypes to soil moisture stress more effectively. To the best of our knowledge, this is perhaps the first report stating the use of a phenomics method as a promising tool for assessing growth rates and also the productive use of water in mungbean crop.

## Introduction

In the human diet, particularly for most of the vegetarian population, mungbean (*Vigna radiata* L. Wilczek), a legume, is an economical and environmentally friendly source of protein (20.97–31.32%) (Yi-Shen et al., 2018). It provides a major amount of proteins (240 g kg^−1^) and carbohydrates (630 g kg^−1^) and a range of micronutrients in diets (Nair et al., 2013). Mungbean, being a short-duration crop (~60–70 days), fits well into different cropping systems dominated by staple food crops such as rice and wheat and the crops broadly cultivated in many Asian countries as well as in the sub-Saharan Africa, dry regions of southern Europe, warmer parts of Canada, and the USA (Nair et al., 2013; Hou et al., 2019). However, this crop in the agricultural landscape is invariably featured by abiotic stresses such as water scarcity, heat stress, salinity, waterlogging, and low soil fertility (Kaur et al., 2015; Bindumadhava et al., 2016). Despite its relatively better stress adaptability than staple cereals, mungbean is vulnerable to the adverse effects of climate change (Sharma et al., 2016). This can be a significant food security concern for countries such as India that often import a large number of pulses to meet their domestic requirement (Reddy, 2013). The productivity of this crop can be enhanced by introgressing tolerance to stresses caused by heat and drought. The exploration of genetic stocks and dissecting traits contributing resilience to water scarcity is crucial (Reynolds et al., 2016; Singh and Singh, 2016).

Mungbean genotypes that cover the ground rapidly with more biomass accumulation (showing early growth vigor) using the residual or stored soil moisture from preceding crops are a target for crop improvement programs (Nair et al., 2019). The crop growth rate estimation based on the plant biomass data at different intervals (Ajlouni et al., 2020) is a challenge for plant breeders while selecting the best out of thousands of progenies, requiring periodic, and destructive sampling (Walter et al., 2015). This process is tedious and expensive, which also pushes us to miss a few promising lines (Montes et al., 2011).

Currently, there is an increasing focus on the traits contributing to drought tolerance and their linkages with genes, which can be introgressed to a desired agronomic background through conventional and/or molecular breeding approaches (Richards et al., 2010; Reynolds et al., 2016). The power of predicting a relationship between the traits and genes can be robust if a large set of genotypes are screened phenotypically for a desired trait or set of traits (Tester and Langridge, 2010). Several traits have been reported to be associated with drought tolerance; however, it is feasible to use a few of them for screening a large number of genotypes by conventional methods (Reynolds et al., 2016). The early growth rate is very critical for the higher water-use efficiency of plants like mungbean at the canopy level as it can help prevent evaporative loss of moisture through the soil surface. High-throughput phenotyping methods are highly essential, particularly for this type of trait.

The solution for the identification of genotypes with such a trait has now emerged in the form of high-throughput plant phenomics tools that are non-destructive, precise, and rapid as they harness the power of multi-dimensional imaging science, information technology, and automation tools (Furbank and Tester, 2011; Al-Tamimi et al., 2016; Chawade et al., 2019; Zhao et al., 2019). Imaging systems sense a different fraction of electromagnetic radiation (EMR) wavelength bands reflected by the plants instantly as well as dynamically in a way to inform their responses to environmental stimuli. These non-destructive phenomics technologies focus on several traits, which, directly or indirectly, reflect chlorophyll content, the plant water content, biomass, and growth potential (Andrade-Sanchez et al., 2014). These technologies have become an integral component of phenotyping platforms, which combine the plant growth in an automated controlled environment with a high-throughput non-invasive imaging to relieve bottlenecks of the phenotype data collection (Al-Tamimi et al., 2016). For precisely extracting the desired information about the plant phenotype from these imaging cameras, phenomics protocols need to be optimized for continuous monitoring of plant growth and its response to environmental stimuli such as soil moisture (Zhao et al., 2019).

Several attempts have been made to optimize the methods for phenotyping the responses of plants to soil moisture deficit (Al-Tamimi et al., 2016). However, the image-based methods for one crop may not apply to others due to a vast diversity in plant architecture across crop species, and the target environments may vary widely too (Stewart and Peterson, 2015). Hence, we conducted this study with a dual objective of optimizing phenomics protocols to identify the mungbean genotypes that accumulate biomass rapidly and to classify them as high and less water-consuming types based on their water-use indices.

## Materials and Methods

### Mungbean Germplasm

In the present study, 24 mungbean genotypes that were earlier evaluated for plant traits such as grain yield, a reaction to mungbean yellow mosaic virus disease (Nair et al., 2017), mineral and phenolic contents (Nair et al., 2015) were chosen, including locally adapted varieties (Supplementary Table 1). Two experiments were conducted to optimize high-throughput phenotyping protocols.

### Plant Growth Conditions

In the first experiment, plants were grown initially under open air (natural) conditions outside a greenhouse in 12-inch-diameter plastic pots (Nisarga 302) filled with 14 kg clay loam soil. Physico-chemical properties of the soil were as follows: pH 8.4, EC 0.24 dSm^−1^, organic carbon 6.3 g kg^−1^, 170 kg nitrogen, 17 kg phosphorous, and 140 kg potash ha^−1^, 72% clay, 24.4% sand, and 4% silt. Eight seeds were sown in each pot, and later only three seedlings were maintained. These pots were shifted to the greenhouse 30 days after sowing. Inside the greenhouse, temperatures were maintained at 32/24°C day/night, 50–65% relative air humidity, and 450–750 μmol m^−2^s^−2^ PAR. Three pots each for well-watered and water-stressed treatment were maintained (as replicates) for each genotype throughout the experiment.

In the second experiment, the growing conditions were almost identical as described above except that the water stress was imposed by depleting soil moisture while keeping the control with the soil moisture level of 60–80% of the field capacity (FC).

### Gravimetric Assessment of Soil FC

Air-dried soil was ground to pass through a 5-mm sieve at room temperature to determine FC. Water holding capacity was assessed by using a gravimetric method (Canavar et al., 2014). Five pots, filled with soil as described in the previous section, were kept in a tray containing water, and the soil was allowed to absorb water through drainage holes at the bottom of the pots by capillary action overnight. The wet surface on the top layer of the soil was considered as an indicator of the completion of capillary action, and hence allowing the absorption of soil moisture up to the FC. Then, excess water was allowed to drain by moving the pots carefully to empty trays without water until there was no sign of water droplets dripping from the pots. The FC was calculated based on the initial dry and final weights of the pots. The pot weights were observed every day, and the reduction in pot weight was used to calculate relative water losses. Nearly 80 and 50% of water at FC were maintained in well-watered and water-stressed treatments, respectively.

### Watering and Weighing

In automated plant phenotyping platforms, plants were watered at the watering/weighing station by using the peristaltic pumps that supply water or nutrient solutions either as a predefined fixed volume or as an individually calculated amount being the difference of a carrier (including pot) weight to a predefined target weight. There were no mineral deposits and algal growth on the soil surface area that could affect the image background quality seen as particle fluorescent signals. During watering (target volume) with a high-throughput system, both weights before and after watering were measured and recorded in the database to estimate the water consumption of plants per day. These values were used for determining a water-use index (WUI).

### Image Acquisition

**Figure 1:**
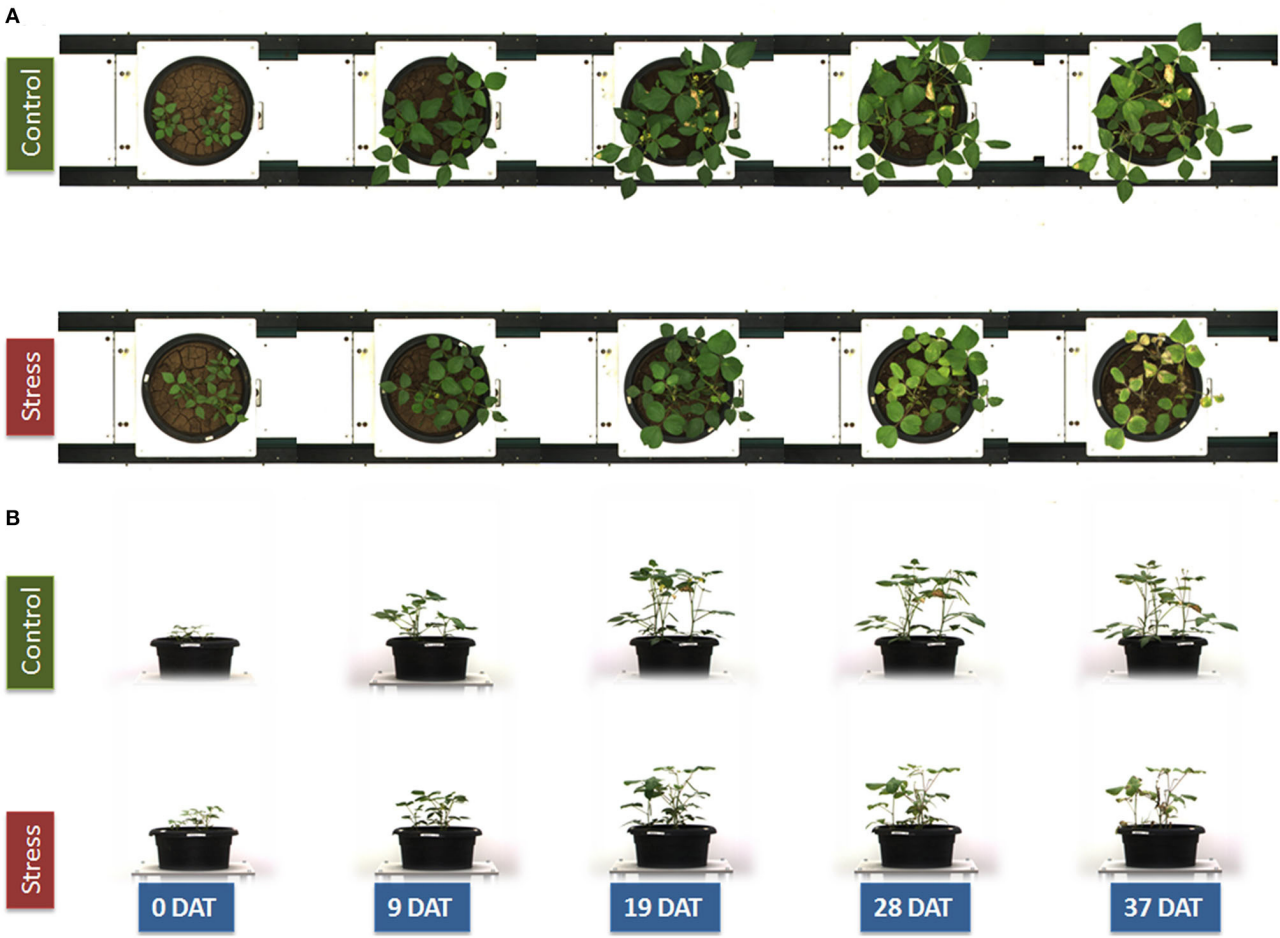
Red green blue (RGB) image of a mungbean genotype IC-415144 on the top view **(A)** and side view **(B)** represents the same plant from 0 to 37 days of control and water stress treatments.

Plants were imaged regularly by using a Scanalyzer 3D imaging system (LemnaTec GmbH, Aachen, Germany). The images of plants were acquired by using light in the visible range of the electromagnetic spectrum, and five-megapixel color images of the plants in each pot were captured in the top view and the side view (Figure 1) at two different rotations (0°, side view 1; 90°, side view 2). High-resolution cameras (piA2400-17gc CCD cameras; Basler, Ahrensburg, Germany) placed at the top and sides of imaging chambers were engaged for capturing the images in the visible range (400–700 nm) of the electromagnetic spectrum. Near-IR (NIR) images in the top view and two side views (0°, side view 1; 90°, side view 2) were acquired by using a NIR-300 camera (NIR-600PGE, Allied Vision Technologies GmbH, Stadtroda, Germany). The camera had a spectral sensitivity of 900–1700 nm and an optical resolution of 320 × 256 pixels. The water absorption band at 1450 nm is the strongest absorption feature in this spectral region, and the NIR signal has been used to estimate the water content in shoots (Seelig et al., 2009). Since the object recognition in the gray-scale NIR images is difficult, the identified object from the red green blue (RGB) images was used to create a mask for overlaying with the NIR images. The mean gray value (8-bit scale) of the identified objects from both NIR images was calculated, where the high values represent a high reflectance and indicate a low water content while the low gray-scale values represent a high absorption and high water content. In addition, IR cameras (IRC-320LGE, Allied Vision Technologies GmbH, Stadtroda, Germany) were engaged to capture the images to interpret the plants' surface temperature.

### Image Analysis and Data Mining

The LemnaGrid software (LemnaTec GmbH, Würselen, Germany) was used for image analysis (Supplementary Figure 1). To get the best segmentation of the plant image from the background, a region of interest was defined to get the entire plant's image, excluding the visible parts of the imaging hardware (e.g., lifter/turner). Plants were segmented from the background by using a nearest-neighbor color classification. The noise was removed through erosion and dilatation steps before composing all parts identified as a plant to one object. Since the plants' height did not vary significantly, there was no need of accounting image pixels for the changing distance effect. The output from the image analysis was then converted into a data table for various image parameters through the Data miner software (LemnaTec GmbH, Würselen, Germany). We generated the data on 18 different parameters (Supplementary Table 2) for each of the three acquired images (top and two side views) for each of the plants. There were 42 different parameters depicting only the plant geometry available to predict the biomass of plants for growth responses to different levels of soil moisture.

### Measurement of Actual Plant Biomass

Plants were harvested 67 days after sowing. The leaf and the stem fresh weights (g) per pot were determined and also on a single-plant basis by harvesting manually the shoot (above ground) using a medium-scale balance (Model Ohaus R21PE30). The sum of leaf and stem fresh weight was considered as the total shoot biomass. Dry weight was recorded after drying the harvested shoot biomass in a hot air oven at 65°C till constant tissue weights were obtained.

### Selection of Biomass Prediction Model

In order to assess plant growth through a time scale, eight different growth models *viz*. linear model (LM), random forest (RF), step model (SM), elastic net, gradient boosting machine (GBM), principal component (PC), partial least square (PLS) regression, and multivariate adaptive regression splines (MARS) were applied by using machine learning algorithms. Actual measured fresh biomass was used as a reference for predicting the biomass from all the geometric parameters of each of the images acquired from the three different views of the plants. The suitability of each model was judged on how well it approximated the data based on a best-fit analysis using mean absolute error (MAE), root mean square error (RMSE), and *R*^2^ values (Tables 1A–C). The following formulae were used for assessing these parameters.

(1) $$\begin{array}{r} \text{MAE}=\text{Actual values}-\text{Predicted values} \end{array}$$

(2) $$\text{MSE =}\frac{\text{1}}{N}\sum_{i=1}^{N}{\left(\text{Actual values}-\text{Predicted values}\right)}^{\text{2}}$$

(3) $$\begin{array}{r} RMSE=\sqrt{\text{MSE}} \end{array}$$

where *N* represents the total number of combinations tested for the validity of the model with relatively higher mean *R*^2^ (R-squared), and less MAE and RMSE were used to finalize the model for the prediction of the biomass of all the plants for all the days. *R*^2^ inevitably increases with additional predictors within one data set. However, a cross-validation error decreases only as long as the additional predictor improves the prediction accuracy of the model in an independent data set (Golzarian et al., 2011). The cross-validation analysis was performed by using the R package “DAAG” (Bay and Schoney, 1982; Rahaman et al., 2017). Based on all these analyses, the PLS was prioritized for the prediction of biomass, which was used to estimate growth rates and a WUI (Supplementary Table 3).

**Table 1:** Validation parameters for different machine learning algorithm-based prediction models employed for predicting fresh biomass.

	**Min**.	**1st Qu**.	**Median**	**Mean**	**3rd Qu**.	**Max**.
**(A) MAE**						
LM	2.45	3.83	4.36	4.46	4.94	8.04
RF	2.24	3.12	3.64	3.69	4.13	5.86
SM	2.32	3.74	4.45	4.33	5.11	6.07
Elasticnet	1.56	2.98	3.54	3.48	4.1	5.27
GBM	2.11	3.2	3.93	3.83	4.49	5.79
PC	1.97	2.85	3.48	3.55	4.09	6.6
PLS	1.74	2.92	3.21	3.44	3.86	6.29
MARS	2.44	3.4	3.92	4.02	4.66	5.93
	**Min**.	**1st Qu**.	**Median**	**Mean**	**3rd Qu**.	**Max**.
**(B) RMSE**						
LM	3.32	5	5.77	5.72	6.34	9.74
RF	2.7	4.2	4.97	5.02	5.86	8.02
SM	2.89	4.98	6.02	5.76	6.65	8.13
Elasticnet	1.95	3.98	4.9	4.68	5.23	7.58
GBM	2.79	4.45	5.37	5.12	5.86	7.73
PC	2.4	3.91	4.87	4.92	5.78	8.42
PLS	2.43	3.78	4.27	4.75	5.51	9.00
MARS	3.02	4.67	5.42	5.49	6.42	8.55
	**Min**.	**1st**	**Qu**.	**Median**	**Mean**	**3rd**
**(C) R squared**						
LM	0.79	0.91	0.94	0.93	0.96	0.98
RF	0.88	0.93	0.95	0.95	0.96	0.99
SM	0.85	0.92	0.93	0.93	0.95	0.99
Elasticnet	0.88	0.93	0.96	0.95	0.98	0.99
GBM	0.89	0.93	0.95	0.95	0.96	0.99
PC	0.84	0.93	0.96	0.95	0.97	0.99
PLS	0.83	0.94	0.96	0.95	0.97	1.00
MARS	0.87	0.92	0.94	0.94	0.96	0.98

### Growth Rates and WUI

Growth rates were calculated for the entire experimental period or at different phases of plant growth by using the following formulae (Williams, 1946).

Absolute Growth Rate (AGR) [gd^−1^]

(4) $$\begin{array}{r} {\text{AGR}}_{\left(tJ,tk\right)}=\frac{{\text{PFB}}_{tk}-{\text{PFB}}_{tj}}{tk-tj} \end{array}$$

Relative Growth Rate (RGR) [gg^−1^d^−1^]

(5) $$\begin{array}{r} {\text{RGR}}_{\left(tJ,tk\right)}=\frac{\ln\left({\text{PFB}}_{tk}\right)-\ln\left({\text{PFB}}_{tj}\right)}{tk-tj} \end{array}$$

where “PFB” refers to the predicted fresh biomass, “*tk*” and “*tj*” are the final day and the initial day, respectively, of the time interval at which the growth rate was computed.

WUI (in mg PFB ml^−1^) was computed by using the following formulae,

(6) $$\begin{array}{r} {\text{WUI \_ BM}}_{tj}=\frac{\text{PFB}}{{\text{WUCum}}_{tj}} \end{array}$$

(7) $$\begin{array}{r} {\text{WUI \_ AGR}}_{\left(tk,tj\right)}=\frac{{\text{AGR}}_{\left(tk,tj\right)}}{{\text{WU}}_{\left(tk,tj\right)}} \end{array}$$

(8) $$\begin{array}{r} {\text{WUI \_ RGR}}_{\left(tk,tj\right)}=\frac{{\text{RGR}}_{\left(tk,tj\right)}}{{\text{WU}}_{\left(tk,tj\right)}} \end{array}$$

where cumulative “WU” refers to the total amount of water utilized for both transpiration and evaporation process during the interval between “*tk*” and “*tj*.” WUCum and PFB refer to the cumulative use of water and the predicted fresh biomass on a particular day. AGR and RGR refer to the absolute growth rate and relative growth rate during the interval between *tk* and *tj*.

### Broad Sense Heritability

Heritability of WUI_RGR was calculated by adopting the formula compiled by Schmidt et al. (2019) and by using the “H2cal” function of “agridat” package in R. The following equations were used for the estimation of heritability (H^2^) by a standard Cullis et al. (1996) and Piepho and Möhring (2007) method, which have been explained in the notes given in Supplementary Table 4.

Standard method

(9) $$\begin{array}{r} H^{2}=\frac{\sigma_{\text{g}}^{2}}{\sigma_{\text{p}}^{2}} \end{array}$$

where ${\;\sigma }_{\text{g}}^{2}$ is the genotypic variance and $\sigma_{\text{p}}^{2}$ is the phenotypic variance.

Cullis method

(10) $$\begin{array}{r} H_{\text{Cullis}}^{2}=1-\frac{v_{\Delta }^{-BLUP}}{2\sigma_{\text{g}}^{2}} \end{array}$$

where ${\;\sigma }_{\text{g}}^{2}$ is the genotypic variance and $v_{\Delta }^{-\text{BLUP}}$ is the mean variance of a difference of two genotypic BLUPs (best linear unbiased predictor of genotype main effects) as explained by Covarrubias-Pazaran (2019).

Piepho and Mohring method

(11) $$\begin{array}{r} H_{\text{Piepho}}^{2}=\frac{\sigma_{\text{g}}^{2}}{\sigma_{\text{g}}^{2}+\frac{v_{\Delta }^{-}BLUE}{2}} \end{array}$$

where $\sigma_{\text{g}}^{2}$ is the genotypic variance and $v_{\Delta }^{-\text{BLUE}}$ is the mean variance of a difference of two genotypic BLUEs (i.e., adjusted means based on best linear unbiased estimators for genotype main effects) as explained by Covarrubias-Pazaran (2019).

Heritability analysis of WUI parameters was carried out with the data generated during the first 3 weeks after imposing water stress by taking into consideration the variances due to replication, treatment (T), days after treatment (DAT), and genotype (G) in addition to an interaction effect between G × T, G × DAT, and G × T × DAT as they all contribute to a phenotypic variance.

### Statistical Analysis

Specific models were selected based on the MAE, RMSE, and R^2^ by using the statistical package “Caret” in R (Kuhn, 2008). We employed the smooth spline function of the Growth Pheno package designed for R (Brien, 2020) to fit a spline to all predicted biomass values. The fitted spline derivatives were obtained, and the AGR and RGR were computed by using them. By default, the smooth spline will issue an error if it is not having at least four distinct *x*-values. On the other hand, fit splines issue a warning and set all smoothed values and derivatives to NA. The handling of missing values in the observations is controlled *via* na.x.action and na.y.action.

ANOVA was carried out with general linear models (GLMs) by using the “agricolae” package in R. Data were tested for normality and log-transformed if necessary to satisfy the assumptions of the statistical methods. The Duncan Multiple Range Test was implemented for pairwise comparisons of means. Any pair of means annotated with the same letter in graphs indicate the absence of a significant difference at 95% CI.

## Results

### Performance Evaluations of the Proposed Model

For the selection and evaluation of the model, we used the “Caret” package of R as it takes information from different machine learning packages such as RF and PCL. It allows nearly identical lines of codes for different models including those selected for the present study (Kuhn, 2008). Further, this automatically resamples the models and conducts parameter tuning. This enables one to build and compare models with very little overhead.

Preferring the features for regression over the classification for prediction, we selected eight machine learning models for the prediction of fresh biomass and evaluated their performance. When there is a sufficient data set, conventionally, the data are split into training and test data sets. However, we used a whole set of corresponding data of image features on the final day of the experiment when the actual biomass was estimated. This set of data was not sufficient to split into training and test data for some of the chosen models. So, instead of splitting the data into training and test data sets, we preferred realistic model estimates through built-in resampling. We used a train function of the Caret package to predict the best regression model and extracted the best model with an attribute “final.model.” The training set up for the resampling had repeated CV as a method, 10 times sampling each with five repeats. The object created by this function was used for the performance evaluation of the models in the prediction of biomass. During this exercise, we considered that the goal of a predictive model is to predict the data, which were never seen before, and hence attention was made to retain the same data structure for modeling data sets in order to develop a model that will predict new data sets.

The resampling approach enabled the computation of realistic *R*^2^ values as it involved subsetting of data and using them repeatedly for prediction as if they are a new set of data. The process of carrying out this exercise over and over again is referred to as resampling, which allows a possible bias and omission of outliers to get the best prediction model. This process provides a realistic *R*^2^ to explain the performance of the model with a new data set and those can also be used for a comparison of models. This is in contrast to the conventional approach of *R*^2^ without sampling, which is not a realistic measure of how well the model is likely to perform on a new data set.

*R*^2^ is the proportion of variation in the outcome that is explained by the predictor variables. In multiple regression models, *R*^2^ corresponds to the squared correlation between the observed outcome values and the predicted values by the model and is often referred to as the coefficient of determination. The higher the *R*^2^, the better is the model. Other measures for the performance of the evaluation of prediction models were RMSE and MAE. RMSE measures the average error performed by the model in predicting the outcome for an observation. Mathematically, the RMSE is the square root of the mean squared error (MSE), which is an average squared difference between the observed actual outcome values and the values predicted by the model. So, the lower the RMSE, the better the model. MAE measures the prediction error. Mathematically, it is an average absolute difference between the observed and predicted outcomes, and hence if MAE is lesser the better the model.

Conventionally, Akaike's information criteria (AIC) and Bayesian information criteria (BIC) are the commonly used unbiased estimate of the model prediction error MSE, a metric developed by the Japanese Statistician, Akaike (1970). The basic idea of AIC is to penalize the inclusion of additional variables to a model. It adds a penalty that increases the error when including additional terms. The lower the AIC, the better the model. On the other hand, BIC is a variant of AIC with a stronger penalty for including additional variables to the model. A prediction model with lower values of AIC and BIC is considered better than the model having higher values for these estimates.

AIC, which is defined as AIC = 2*k*−2ln(*L*), can be employed for the evaluation of the accuracy of multivariate models like linear multiple regression, where the number of parameters (*k*) is actually the number of predictors used in the multivariate regression. In addition, AIC computation requires ln(*L*), which is a log-likelihood possibly for multivariate LMs. In contrast, the multivariate computation that involves RF is not fitted by using maximum likelihood and there is no obvious likelihood function for it. The second problem is the number of parameters *k*, for RF being not clear and any of the parameters such as number of trees, their depth, number of splits if used in the computation of AIC, can be misleading. Hence, for evaluation and comparison, we used the features of Caret package (Kuhn, 2008) that enables the computation of the realistic MSE, RMSE, and *R*^2^ values by bootstrap resampling with 25 repetitions—this is the default resampling approach in caret. In our study, there was a marginal difference in the generally computed, but an unrealistic, *R*^2^ value, and the realistic *R*^2^ values were extracted from resampling.

The influence of a number of randomly selected predictors on the performance of the model is one of the concerns in arriving at the best performing model, but the best value cannot be derived analytically and will be different with different data. Hence, to get the best performance model for each of the machine learning algorithms, the inbuilt features were used for tuning parameters, and the best performing models within each of the machine learning algorithms were used for final comparisons.

**Figure 2:**
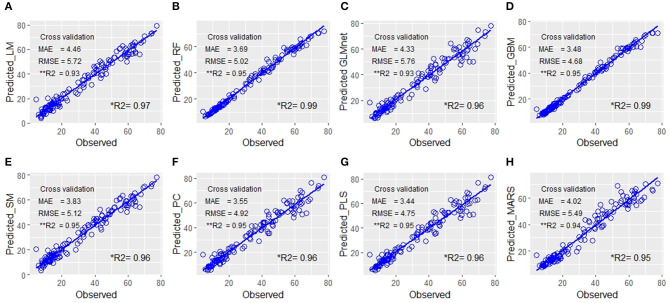
Quantitative relationship between the predicted fresh biomass (PFB) from image-features and the observed plant fresh biomass. Scatter plots of the manually measured plant biomass (fresh weight) vs. predicted biomass values using eight prediction models: **(A)** linear model (LM), **(B)** random forest RF, **(C)** GLMnet, **(D)** gradient boosting machine (GBM), **(E)** step model (SM), **(F)** principal component (PC), **(G)** partial least square (PLS), and **(H)** multivariate adaptive regression splines (MARS). The blue line indicates the expected prediction (*y* = *x*). The prediction models were evaluated by mean absolute error (MAE), root mean square error (RMSE), and the percentage of variance explained by the models (the coefficient of determination *R*^2^). Each figure in the panel has **R^2^ values computed as the mean of *R*^2^ of repeated sampling of data and is a more realistic estimate of power of prediction of the model with a new set of data in comparison to the conventional *R^2^ value derived from the regression equation using a whole set of predicted values without resampling.

For the reason explained above, we preferred the most widely used performance evaluation metrics of MSE, RMSE, and *R*^2^ values along with their statistical parameters (mean, median, and quartiles) computed for all the samples of each of the eight models viz. LM, RF, stepwise regression model (SM), GLMnet, GBM, PC analysis, PLS regression, and MARS for the prediction of biomass. The Results revealed that PC and PLS were relatively more efficient with relatively low values for MAE and RMSE and high *R*^2^ values (Table 1, Figure 2). Though the RMSE values of GLMnet were relatively lower, the range was wider than PC or PLS. However, we preferred PLS over PC as a cross-validation revealed that the least RMSE could be achieved with a minimum number of components relative to the latter model (Supplementary Figure 2). In addition, 93% of the variance was explained by only three components in PLS while PC used nine components.

**Figure 3:**
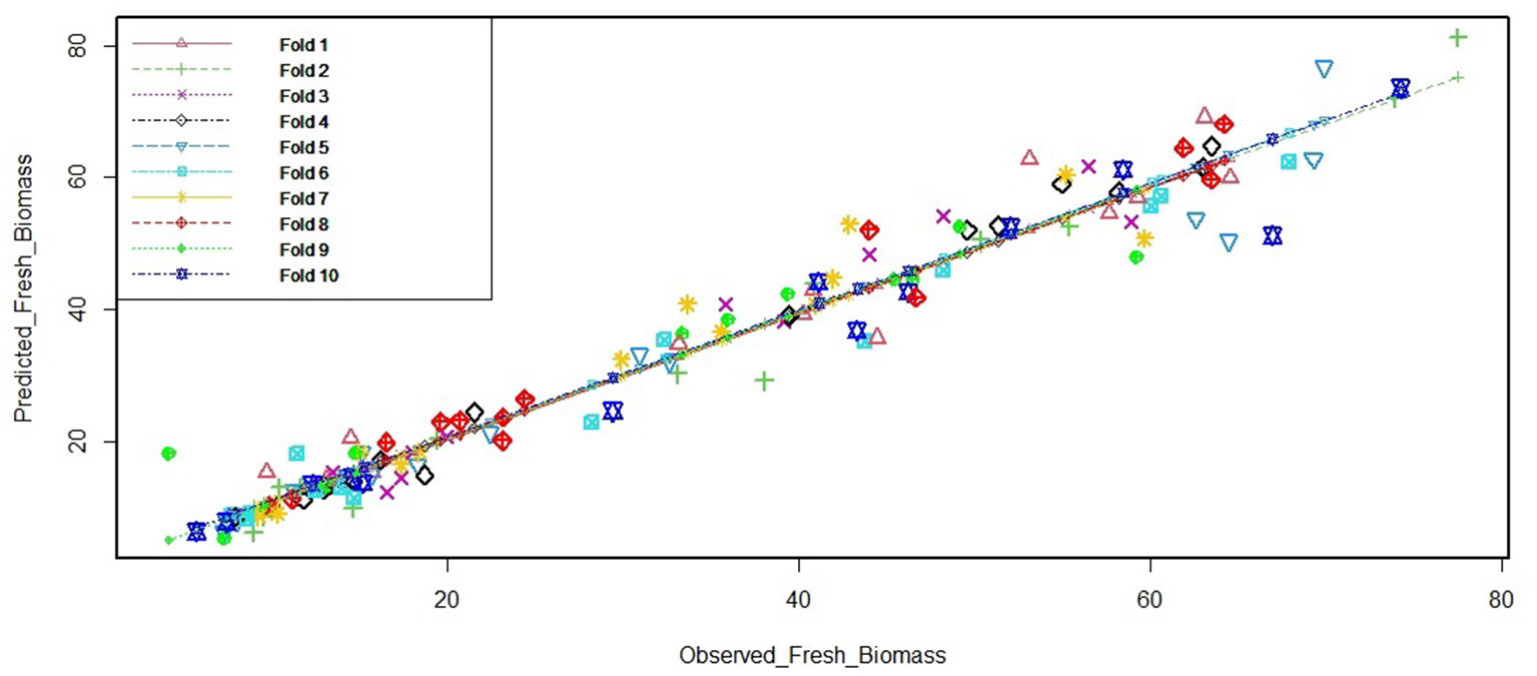
Cross-validation output for the final PLS model. The “cv.lm” function of “DAAG” package was used for simple linear regression models. The *k*-fold method randomly removes *k*-folds for the testing set and models the remaining (training set) data. Here, we use the commonly accepted 10-fold application. The figure depicts the cross-validation residual sums of squares, which is a corrected measure of prediction error averaged across all folds. The function also produces a plot of each fold's predicted values against the actual outcome variable (y); each fold having a different color.

When the non-availability of test data makes the estimation of test error very difficult, the situation is handled by methods such as cross-validation (Varoquaux et al., 2017) that is applied for estimating the test error (or the prediction error rate) by using training data. As explained earlier in this section, the model for prediction was built based on the cross-validation options. In addition, as a double check of accuracy of the PLS model finally selected for further use in the experiment, we performed a 10-fold cross-validation (Figure 3) using the cv.lm function of “DAAG” package of R (Bay and Schoney, 1982). The cv.lm function gives internal and cross-validation measures of predictive accuracy for ordinary linear regression. The data are randomly assigned to a number of “folds.” Each fold is removed in turn while the remaining data are used to refit the regression model and to predict at the deleted observations. The 10-fold cross-validation was used to assess the prediction performance of the final PLS model. The ANOVA test revealed that the prediction of fresh weight by the finalized model was highly significant (*p* < 2e-16). Hence, we chose a PLS model for the non-destructive estimation of biomass of plants from the day of stress treatment.

### Biomass and Growth Rate

**Figure 4:**
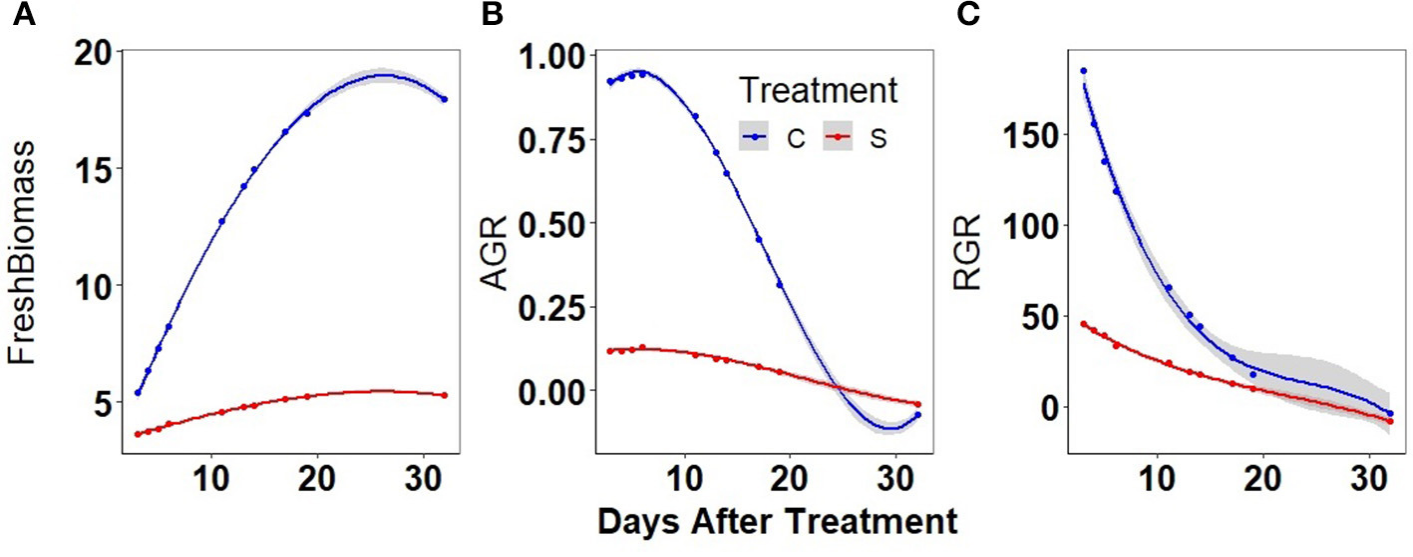
Biomass and absolute growth rates (AGRs) in well-watered (C) and water-stressed (S): mungbean plants: **(A)** biomass was predicted by PLS based on different image parameters, **(B)** smoothed AGR values were obtained from the predicted biomass, and **(C)** smoothed relative growth rate (RGR) values were obtained from the predicted biomass. The solid line represents the grand average of well-watered conditions (blue) and water-stressed conditions (red). Shaded part along the curve displays CI of 0.95.

It was observed that the predicted biomass did increase continuously in well-watered plants while it was nearly stagnant in water-stressed plants. Approximately a 4-fold increase in biomass was observed at the end of the experiment in well-watered plants compared to a nearly 2-fold increase in water-stressed plants (Figure 4A). Similarly, the AGR showed significant differences in well-watered (4-fold increase) and water-stressed plants at initial stages (Figure 4B). The RGR differences between the treatments were significant in the first week of water stress (Figure 4C).

### Genotypic Variation in Growth Rates

**Figure 5:**
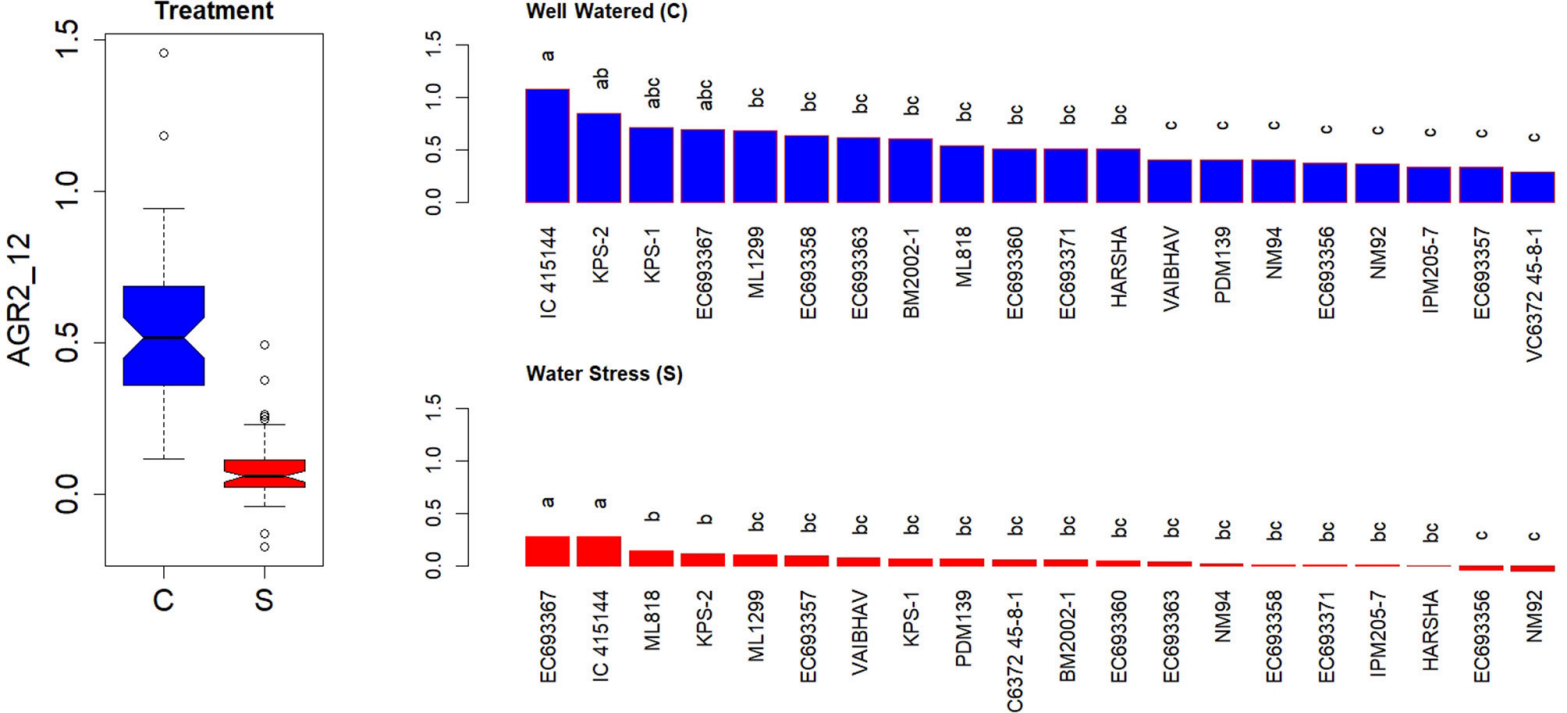
Genetic variation in AGRs during the initial phase in response to two levels in well-watered (C) and water-stressed (S) plants; AGRs were computed for 2–12 days. Each box in the treatment effect represents 60 observations (3 replications and 20 genotypes). Each bar in the genotype effect for each treatment represents mean values of three replications. Letters represent the significance of differences among mean values as computed by the Duncan multiple range test at 0.95 CI. Genotypes with common letters are not significantly different.

The measured AGR during the first 6 DAT revealed a significant genotypic variation across water-stressed than well-watered conditions. The average values for AGR at the initial phase of the stress were more than 6-fold (Supplementary Figure 3A). A similar trend was observed in the computed AGR for the interval between 5 and 12 days after the stress (Supplementary Figure 3B) or during the interval between 2 and 12 days after the stress (Figure 5). Genotypes such as EC 693367 and IC-415144 maintained higher AGR in contrast to any other genotypes under soil moisture stress conditions during the period between 2 and 12 days after stress.

**Figure 6:**
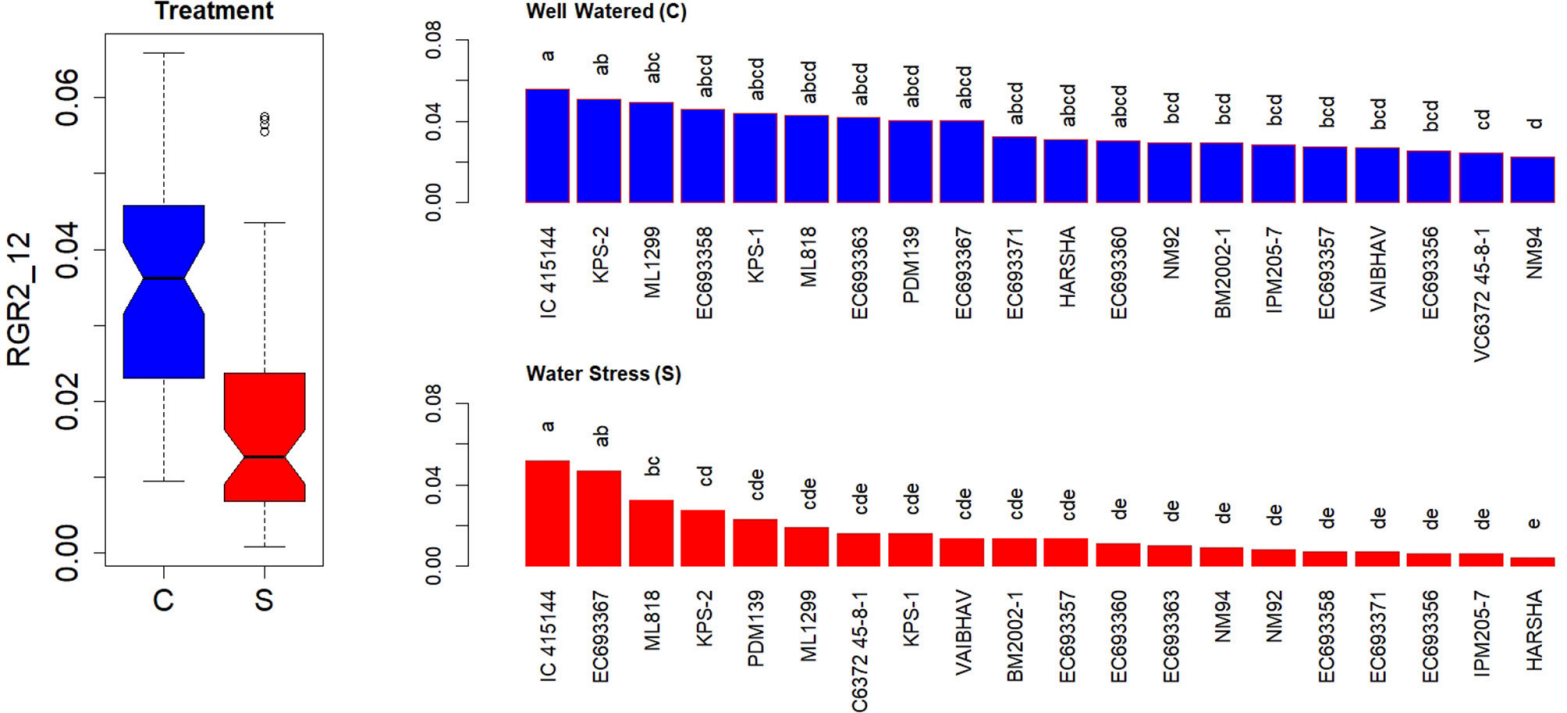
Genetic variation in RGRs during the initial phase in response to two levels in well-watered (C) and water-stressed (S) plants; RGRs were computed for 2–12 days. Each box in the treatment effect represents 60 observations (3 replications and 20 genotypes). Each bar in the genotype effect for each treatment represents mean values of three replications. Letters represent the significance of differences among mean values as computed by the Duncan multiple range test at 0.95 CI. Genotypes with common letters are not significantly different.

In contrast to AGR, RGR showed a significant genotypic variation both under well-watered and water-stressed conditions at the early stages after the stress treatment (2–6 days) (Supplementary Figure 4A). However, the contrast between the genotypes was widened and became more conspicuous at later stages (5–12 days) (Supplementary Figure 4B). RGR measured for the first 3 weeks after the stress treatment could also differentiate the genotypic responses more efficiently (Figure 6). EC 693367 and IC-415144 maintained higher RGR in contrast to any other genotypes under soil moisture stress conditions during the period between 2 and 12 days after stress.

### Water-Use Index

**Figure 7:**
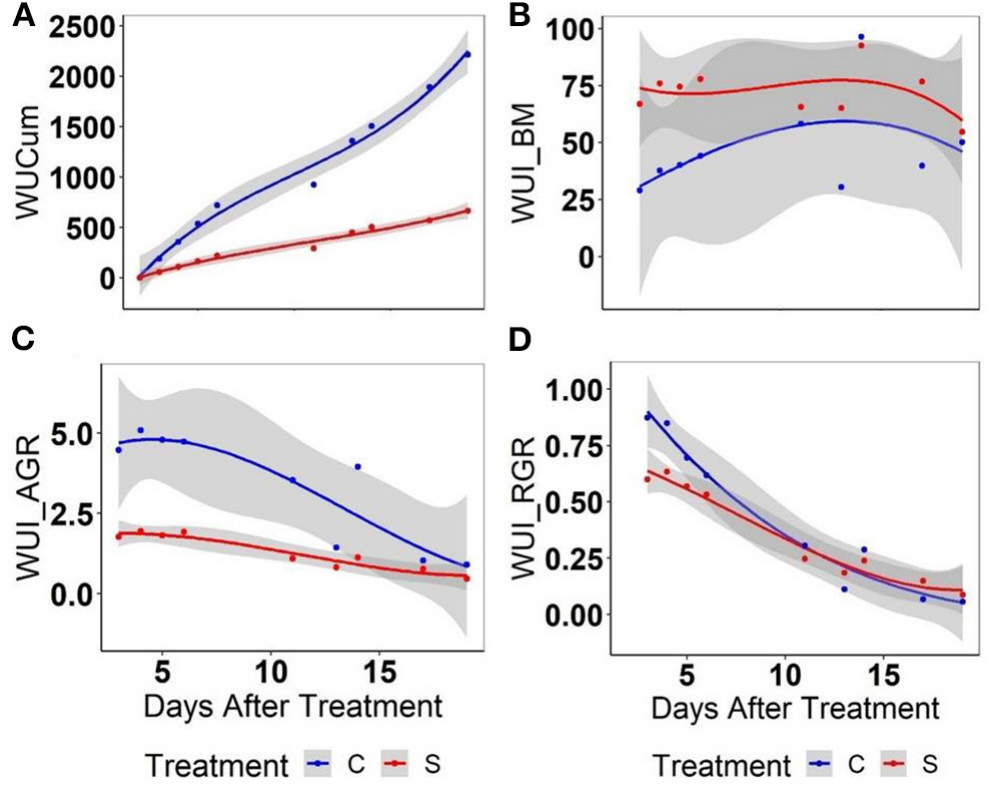
Assessment of different parameters for water use using mungbean plants: **(A)** cumulative water use (WUCum), **(B)** a biomass-based water-use index (WU_BM), **(C)** an AGR-based water-use index, and **(D)** a RGR-based water-use index in well-watered (C) and water-stressed (S) plants: WUCum represents the cumulative water use in ml plant^−1^ at any given point of growth while WUI_BM computed as the predicted biomass mg ml^−1^ water, WUI_AGR or WUI_RGR was computed for per unit of water as given in Material and Methods. Solid lines represent the means of all genotypes while the shaded part indicates the CI at 0.95.

As expected, cumulative water use (WUCum) was 4-fold higher in well-watered relative to water-stressed plants 3 weeks after imposing the treatment (Figure 7A). In contrast, the WUI (WUI_BM) representing the biomass accumulation per unit of spent water was high in water-stressed relative to well-watered plants but the treatment difference was not significant (Figure 7B). In contrast, WUI_AGR computed based on AGR (Figure 7C) could differentiate the plant responses to water stress more effectively as compared to the WUI_RGR computed based on the RGR (Figure 7D), particularly at the early stage after water stress. With the lapse of time, WUI of both well-watered and the water-stressed plants were found to be the same. Considering this fact, the genetic variation for WUI was assessed for the initial 6 days of treatment only.

### Genetic Variation in WUI and Tissue Water Content (Derived From NIR Reflectance)

**Figure 8:**
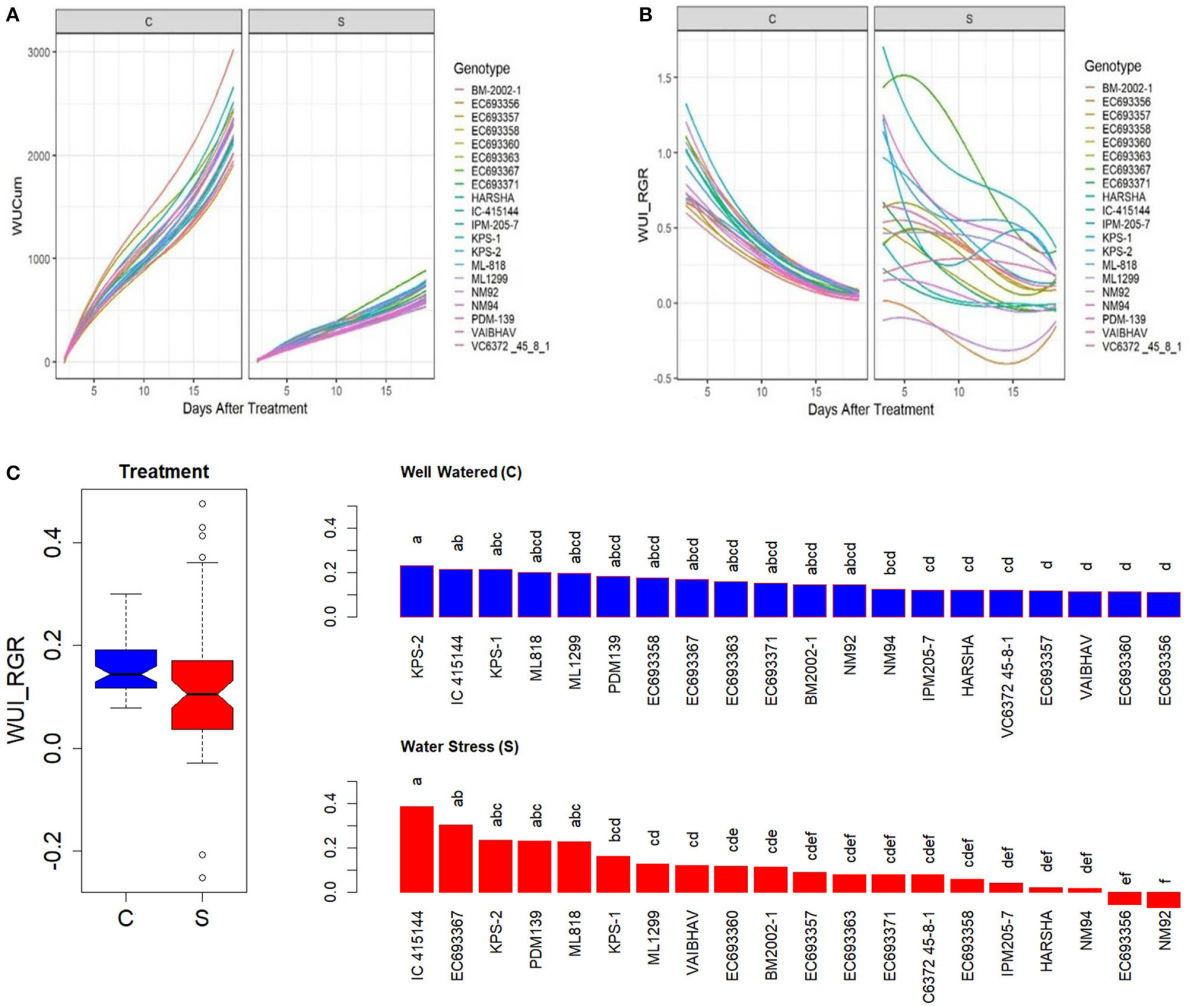
Genetic variation in **(A)** cumulative water use (WUCum, ml plant^−1^), **(B)** WUI_RGR derived from RGR as in Material and Methods, and **(C)** genetic variation in WUI_RGR of mungbean genotypes under well-watered (C) and water-stressed (S) conditions. In **(C)**, the values for each box in the treatment effect represent 60 observations (3 replications and 20 genotypes), and each bar in the genotype effect for each treatment represents mean values of three replications. Letters represent the significance of differences among mean values as computed by the Duncan multiple range test at 0.95 CI. Genotypes with common letters are not significantly different.

There was a significant variation in cumulative water-use WUCum among the genotypes both under well-watered and water-stressed conditions (Figure 8A); however, the genotypic differences in the trend of drought responses were more conspicuous in WUI_RGR (Figure 8B). Under limited irrigation, genotypes such as IC-415144, EC693367 had high WUI_RGR relative to genotypes such as NM92 and EC693358 (Figure 8C).

**Figure 9:**
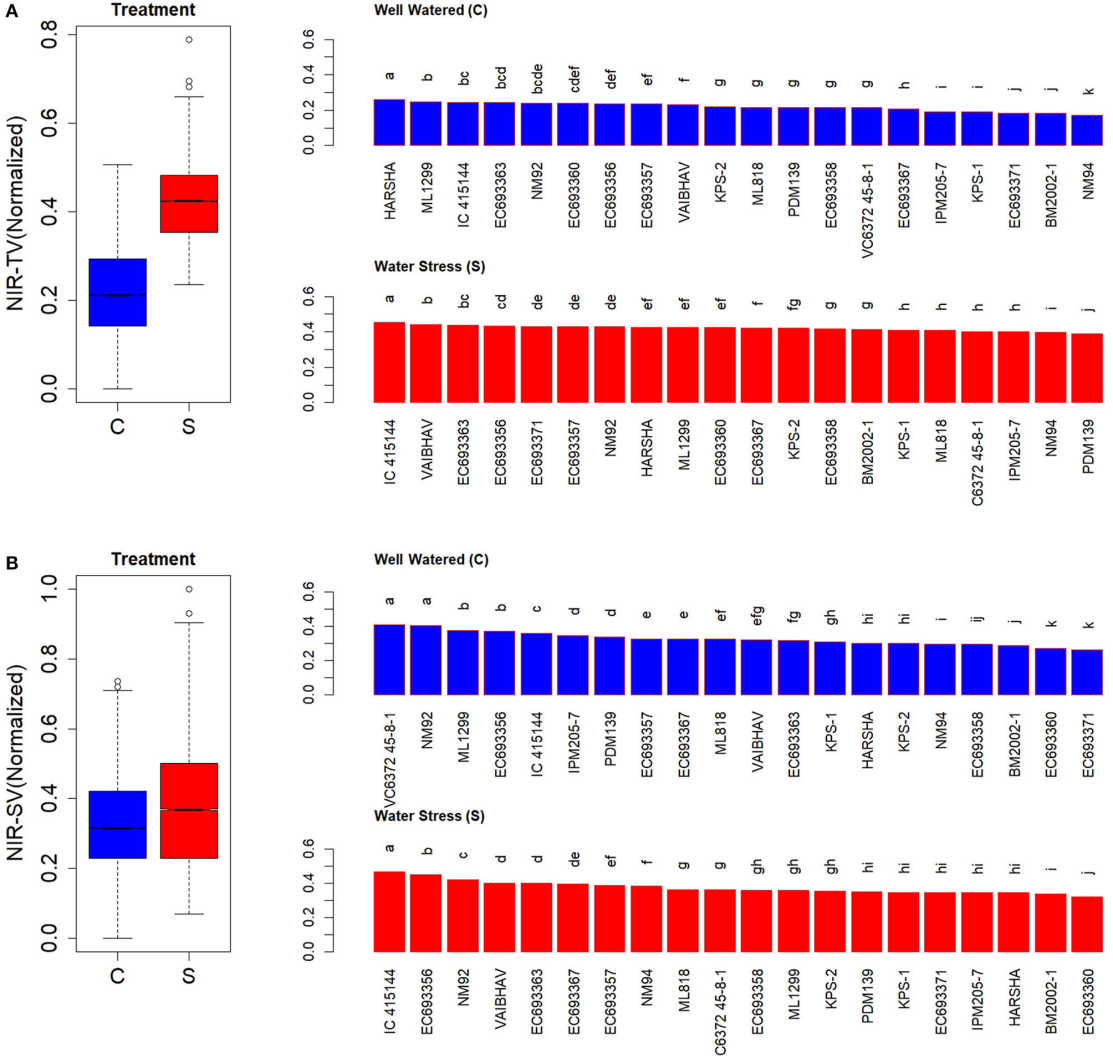
Genetic variation in the tissue water content of mung bean genotypes as indicated by near-IR (NIR) signals. **(A)** Values indicate normalized values of NIR signals received from the top view and **(B)** side view of well-watered (c) and water-stressed (s) plants of mungbean. Values for each box in the treatment effect represent 60 observations (3 replications and 20 genotypes) and each bar in the genotype effect for each treatment represents mean values of three replications. Letters represent the significance of differences among mean values as computed by the Duncan multiple range test at 0.95 CI. Genotypes with common letters are not significantly different.

There were significant differences in tissue water contents among the genotypes studied in this experiment, as reflected by NIR signals received from the top view and the side view (Figure 9). NIR signals tend to be higher when emitted from dry leaves than from wet leaves due to the absorption of EMR in the NIR range of the spectrum. The results from the present experiment revealed that the high WUI_RGR genotypes such as IC-415144 and EC693367 had less tissue water content compared to EC693360, NM94, and BM2002-1, which had relatively low WUI_RGR under water-stressed conditions. Further, it was evident that the genotypes like IC-415144 had relatively less tissue water content compared to other genotypes both under well-watered as well as water-stressed conditions providing a hint that it must be extracting water from soil with greater efficiency.

**Figure 10:**
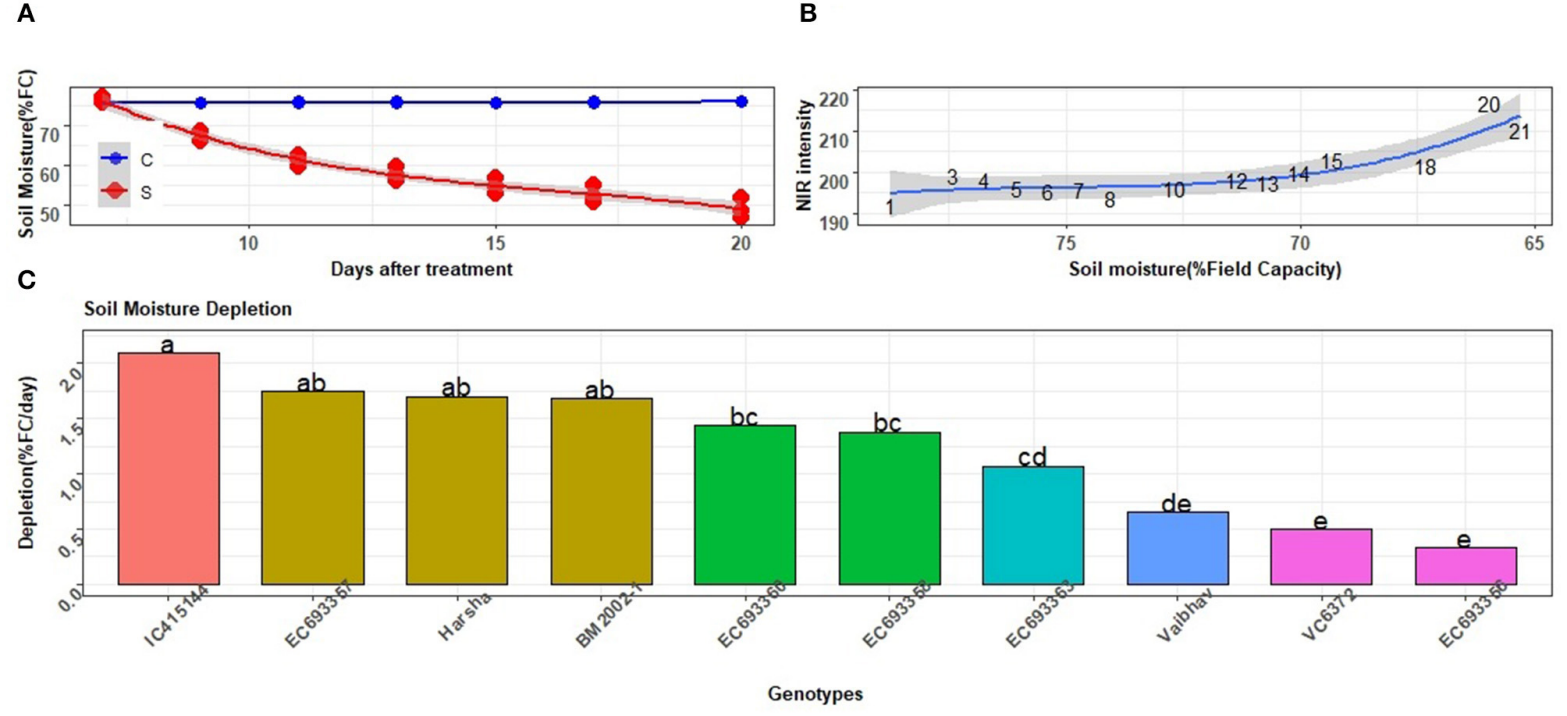
Responses of the selected mungbean genotypes to depleting soil moisture. **(A)** Level of soil moisture as indicted by % field capacity (FC) in well-watered (c) and water-stressed (s) plants. Solid lines represent mean values for all genotypes in each of the treatments. **(B)** Tissue water content in response to the depletion of soil moisture as indicated by NIR signals. High and low NIR values indicated more and less tissue water content, respectively. Values indicated along the curve represent days after withdrawal of water application to pots. **(C)** Rate of depletion of soil moisture in different mungbean genotypes; each value is an average of three replications of water-stressed plants. Letters represent the significance of differences among the mean values as computed by the Duncan multiple range test at 0.95 CI. Genotypes with common letters are not significantly different.

Compared to the constant level of soil moisture stress, depletion of the soil moisture condition represents open field environments that face unexpected drought or limited irrigation. The level of depletion could easily be monitored in a high-throughput mode for all genotypes. The stress imposed in the second experiment did result in the depletion of soil moisture from 70 to <50% of the FC while the same was around to be constantly 75% in well-watered plants (Figure 10A). The impact of imposed water stress was also reflected by the NIR signals that increased from about 180 to 220 with a gradual reduction in soil moisture (Figure 10B). There was a significant difference among genotypes in their capacity to extract water from the soil (Figure 10C). Genotypes such as IC-415144, which had high WUI and drier leaves in the previous experiment, found to deplete more soil moisture relative to other genotypes, thus substantiating the results of the previous experiment.

### Heritability of WUI

Heritability in a broad sense was computed for WUI derived from fresh biomass, AGR, or RGR at the early stages of stress. Heritability of traits WUI_AGR or WUI_RGR was higher than that of WUI_BM. This could be attributed to the relatively higher heritability of AGR and RGR at the early stages of water stress (Supplementary Table 4).

## Discussion

Drought tolerance, a complex plant trait, is highly influenced by the time of occurrence, intensity, duration, and the plant growth stage (Araus et al., 2012; Passioura, 2012). The drought stress experienced by a mungbean crop is typical as it has to survive on the stored or residual soil moisture in the later stages of growth with the luxury of wetness in the early stages (Bindumadhava et al., 2016; Sharma et al., 2016). Further, being a very short-duration crop, a rapid growth at the early stages is crucial for gaining biomass enough to feed all reproductive-stage needs. Hence, the assessment of growth rate assumes to be more significant than biomass at any given crop stage and/or time. Such approaches have been demonstrated to assess the plant growth in crops like barley (Chen et al., 2014; Neumann et al., 2015), rice (Hairmansis et al., 2014; Yang et al., 2014; Campbell et al., 2015; Al-Tamimi et al., 2016), wheat (Nagel et al., 2015), and model crops like *Arabidopsis* (Zhang et al., 2012; Slovak et al., 2014). Some of these studies had also focused on the methods to assess water-use efficiency in crop plants (Al-Tamimi et al., 2016). However, so far, to the best of our knowledge, no high-throughput protocol reported on the assessment of growth rates in legumes like mungbean. Hence, the present study was designed and aimed as a more practical approach toward optimizing the phenotyping method in mungbean genotypes for assessing growth rates and a WUI under controlled drought conditions.

### Determination of Plant Biomass

Most of the conventional studies rely on the determination of plant biomass by a destructive sampling method, which is resource intensive as demonstrated in rice (Yang et al., 2014), wheat (Golzarian et al., 2011), and other annual and perennial grass species (Tackenberg, 2007), and the measurement of water use in a single plant is highly cumbersome. Despite their high accuracy, these methods have limitations in integrating dynamic nature of plant responses in different stress environments.

Further, destructive and periodic sampling does not permit the collection/harvesting of seeds from individual lines tested in crop breeding programs, as seen in rice (Hairmansis et al., 2014). Hence, the focus of the present study has been on using the imaging-based non-destructive approach to predict the plant biomass without a periodic sampling of the whole plant. Image-based biomass estimation methods developed so far used the total biomass at the end of the experiment or the biomass estimated during the measurement of other traits such as shoot fresh weight and shoot dry weight as well (Tackenberg, 2007; Golzarian et al., 2011).

Several methods were suggested earlier to estimate biomass non-destructively and were based on the area of color pixels derived from an analysis of the images of plants from three different views in barley (Neumann et al., 2015) and rice (Al-Tamimi et al., 2016). To further improve the accuracy of the biomass estimation in cereals, an additional parameter such as compactness derived from the images was also suggested (Rahaman et al., 2017). However, we observed that these models have prediction errors when extending to legume crops such as mungbean. Hence, eight different multivariate analysis models were tested with machine learning algorithms to predict the plant biomass, including several image parameters across three different views of plants as predictors. Based on MSE, RMSE, and *R*^2^ values, both PC and PLS methods were relatively more efficient than other models in the prediction of biomass (Table 1). We finally chose a PLS method for the plant biomass prediction as it did not differ significantly from the PC analysis. The choice of the PLS method was further supported by cross-validation of the predicted and actual fresh biomass recorded at the end of the experiment. The predicted biomass showed a clear trend when plotted against days after the water stress treatment with the growth almost stationary in stressed plants and continuous in well-watered plants, which matched with general anticipation. After the confirmation of the precision of the selected model through a robust validation tool, the biomass computed for each day was used for the estimation of growth rates. A similar approach was followed for rice to assess salinity tolerance (Al-Tamimi et al., 2016).

### Plant Growth Rate

The initial growth rate (in place of biomass) is a good measure of the capacity of plants to cope with soil moisture limitations. Some plants may continue their growth by acquiring water efficiently while others may stall the growth during water stress and then recover after the retrieval of stress. The growth rate assessment can differentiate these two groups of plants (Condon et al., 2004; Lopes et al., 2011). In our study, AGR and RGR were considered for assessing the effectiveness of treatments and the effect of genotypes as demonstrated earlier in rice on salt tolerance (Al-Tamimi et al., 2016). As evident from the results, there were significant treatment effects on AGR and RGR computed for two phases of the early growth of mungbean. However, genetic variations in AGR, as well as RGR, were more conspicuous in water-stressed plants. This approach could differentiate the genotypes of mungbean capable of maintaining a relatively higher growth even with limited soil moisture. This feature is crucial for short-duration crops like mungbean, which are accommodated between the two major consecutive crops (mostly cereals) in the commonly practiced cropping (cereal-legume-cereal) system to make the best use of time, space, and moisture leftover in the soil after the harvest of the previous crop, mostly a cereal (Raina et al., 2019).

### Water-Use Index

In this study, we derived and projected water-use efficiency through WUI, a ratio of biomass accumulated to water use (both transpiration by plant and evaporation from the soil surface), often projected as a trait for the improvement of crop productivity in water-deficit agroecologies. Implicitly, crop genotype/s that show more biomass per unit of water can be promising candidates in drought research for developing types of tolerance. Transpiration combines the plant's physiological and environmental parameters that determine water relations in plants (Sinclair, 2012). Hence, it is emphasized that component traits contributing to transpiration efficiency need to be investigated to improve the effective use of available water through the growing season for maximizing the growth and productivity of crop plants. In this context, our focus was on plant trait—early ground cover—that defines the capacity of plants to protect the root zone soil moisture by preventing direct exposure to sunlight. Soil moisture saved in this process can be made available for transpiration to improve water-use efficiency (Raina et al., 2016).

Genotypes that accumulate more biomass can be identified by assessing the growth rates while the water-use estimation is a tedious task. High-throughput phenomics methods are rightly equipped with systems to monitor and replenish the water requirements of several plants regularly. This provision has frequently been used to create the desired level of water stress and assess the water use of crop plants' genotypes (Al-Tamimi et al., 2016). Assessing water-use indices at various water regimes and growing scenarios helps understand the cause-effect relationship between the biomass accumulation and the extent of water use (Sheshshayee et al., 2003). It is also evident that the interrelationship between cumulative water transpired to the biomass production is not always straightforward, which largely depends on whether mesophyll or stomatal factors drive the process (Udayakumar et al., 1998; Sheshshayee et al., 2003; Bindumadhava et al., 2005, 2011). From this background, too, WUI holds a larger significance than measuring WUE either at a single-plant level and/or canopy level (Sheshshayee et al., 2003; Bindumadhava et al., 2011). Hence, we explored different ways to derive WUI for differentiating mungbean genotypes for their water-use efficiency at the canopy level. We imply that measuring WUI non-invasively with the phenomics approach is highly effective in deducing the factors that determine plant biomass and water use. In our study, there was a remarkable difference in the cumulative water use as well as biomass accumulation between well-watered and water-stressed plants. The data revealed that mungbean genotypes, in general, maintain their capacity to use water efficiently even with a limited water supply, as evident from a non-significant difference in RGR between well-watered and water-stressed plants at the later stages of growth. On the other hand, WUI computed as the ratio of AGR to actual water use could differentiate the treatment effects at the initial phases of imposed water stress. Further, these indices also clearly differentiate the genotypes with respect to their capacity to use water efficiently for maintaining growth.

Heritability of traits is an issue when a novel trait is suggested for use in a breeding program for the improvement of crops. The broad sense heritability (*H*^2^) of a trait is the proportion of an overall phenotypic variance attributable to a genotype (Schmidt et al., 2019). It is a descriptive measure to assess the utility and precision of the results obtained from the cultivar responses in trials. Hence, *H*^2^ values were computed for growth rate and WUI indices derived in this study. Reasonably high values of *H*^2^ for WUI_AGR and WUI_RGR provide evidence for suggesting these two parameters as excellent surrogate traits to distinguish the responses of mungbean genotypes to a suboptimal level of soil moisture and interpret genotypic variations for the productive use of water. This can be attributed to the fact that the growth rate can evade the error due to the initial differences in biomass. Here, we could also test the hypothesis that the genotypes with a low WUI probably deplete more soil moisture relative to other genotypes with a high WUI. This was confirmed from our second validation experiment in which the stressed plants were grown by depleting soil moisture in contrast to the first experiment in which the soil moisture was restricted at a suboptimal level. Additionally, the NIR signals revealed that the genotypes having a higher WUI tend to be drier than those with less WUI when the mean values of these parameters for the first 3 weeks after the water stress were considered. It indicates that these genotypes possibly need more water and deplete soil moisture rapidly. Hence, high WUI genotypes might be having an efficient root system, and it needs to be examined further for using genotypes with this trait as donor lines in the mungbean improvement program.

### Relevance of Growth Rates to Field Performance of Mungbean Genotypes

Many of the genotypes included in the present study were examined earlier for their seed yield under a prevailing high temperature environment due to delayed sowing (Sharma et al., 2016; Bindumadhava et al., 2018). In these studies, genotypes such as EC693357, EC693358, Harsha, and ML1299 were found to be heat tolerant, and KPS1, EC693363, NM92, and VC6372 were sensitive. Of several abiotic constraints, high temperature, and drought stresses go hand-in-hand, implying an increase in air temperatures tending to heat up soil temperatures, thereby depleting soil moisture (Bindumadhava et al., 2016; Priya et al., 2019; Douglas et al., 2020). Any crop/plant genotypes withstand high temperatures through organizing or reorganizing intrinsic cellular mechanisms, and could manage soil moisture stress better (Bindumadhava et al., 2016; Nair et al., 2019; Douglas et al., 2020). There is also a consensus that drought-sensitive genotypes experience a sense of high temperature stress when water is withdrawn from their stress thresholds (Sheshshayee et al., 2003; Bindumadhava et al., 2016; Douglas et al., 2020). Similarly, the genotypes that perform well under the soil moisture depletion can handle high temperature stress effectively as a strategic synergy at molecular and gene levels, which have been amply suggested and demonstrated (Bindumadhava et al., 2016; Priya et al., 2019; Chaudhary et al., 2020). Interestingly, EC693357, EC693358, and ML1299 showed salt tolerance both at a seedling and also whole plant level (Manasa et al., 2017). It has been observed that drought-tolerant genotypes of legume crops such as chickpea can tolerate the drought and heat more effectively as compared to those, which could tolerate only the high temperature (Awasthi et al., 2017). These observations all together support the interpretation that the method developed by us to identify promising genotypes being relevant to field performance.

Our data revealed that the AGRs of EC693357 (0.14), ML1299 (0.10), and EC693358 (0.09) were 3- to 4-fold higher than those in sensitive genotypes, such as VC6372 (0.03) and NM92 (0.022) during the initial phases of water stress. Similarly, most of the high temperature-tolerant genotypes had a higher biomass production per unit of water relative to the sensitive genotypes such as VC6372 and NM92. Further, WU_RGR recorded in NM92 was substantially lesser than that of the high temperature-tolerant lines; however, KPS1, which was reported to be sensitive in field studies, also had high WU_RGR. It is suggested that the superior genotypes such as EC693367 and IC-415144 with higher initial growth rates than the promising genotypes mentioned above can also be useful as donors in the genetic improvement of mungbean for drought and high temperature stresses, which is largely due to their rapid growth with a higher extraction or utilization of available soil moisture. Possibly, the higher capacity of evaporative cooling reflected indirectly from higher depletion (or higher transpiration) of soil moisture in these genotypes, particularly at the reproductive stage, might be contributing to the cooler canopy, as shown in the present study, but needs further investigation. IC-415144 is one of the recent collections from arid regions of Rajasthan, India, and has not been studied extensively. In a previous study (Raina et al., 2016), IC-415144 exhibited a low excised leaf water loss and appeared to have a better control over stomatal mechanisms, which might be contributing to high water-use indices. In the present study, WUI_AGR and WUI_RGR could efficiently differentiate genotypes that use more water than others for producing the same amount of biomass. Moderate-to-high heritability recorded for WUI_AGR and WUI_RGR using different methods support the use of these traits as surrogates for efficient selection in mungbean breeding programs.

### Suggested Screening Protocol for Early Growth Rate and Water Use in Mungbean

**Figure 11:**
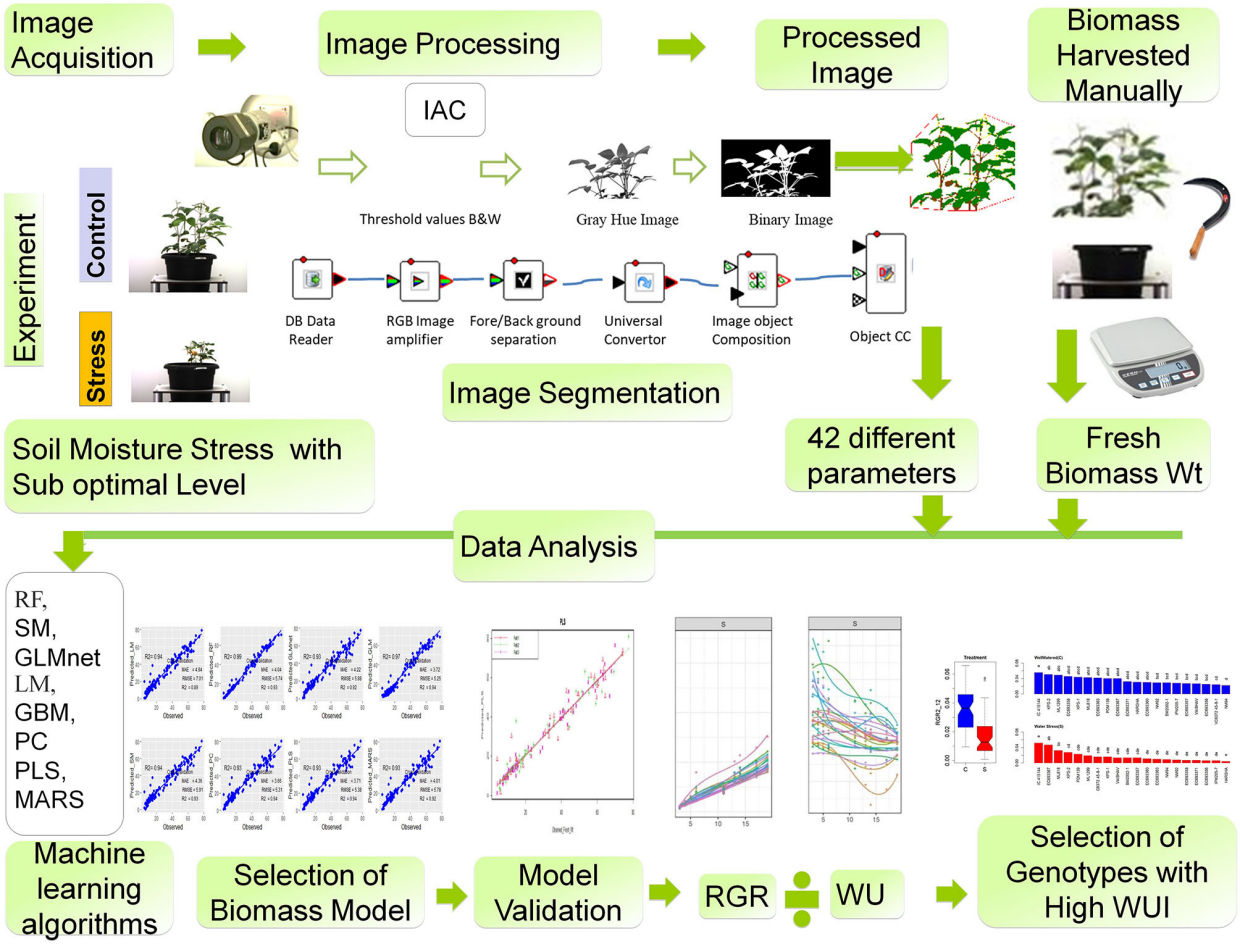
A suggested phenotyping protocol for mungbean genotypes. Workflow for image-derived biomass model construction consists of the following steps: (1) image acquisition, (2) image processing, (3) different geometric and color-related phenotypic trait observed, (4) biomass harvested manually at the last day of imaging and fresh weight and dry weight measured, (5) different machine learning algorithms RF, SM, GLMnet, LM, GBM, PC, PLS, MARS models were used to predict plant biomass, (6) model validation, (7) model selection, evaluation, and result interpretation, and (8) selection of genotypes with high water-use index (WUI).

If the canopy level water-use efficiency trait is a strategy to harvest more grains from legume crops like mungbean, an early growth rate with a high biomass accumulation can serve as a reliable selection criterion. Phenotyping this trait in a high-throughput mode can be effective if the non-destructive estimation of biomass is derived from the changes in plant features that can be extracted from the images captured periodically (Figure 11). Based on our study, we propose a high-throughput phenotyping protocol for mungbean that should involve: (1) the imposition of suboptimal soil moisture stress, (2) acquisition of different parameters of images of plants from the top view and side view, (3) identification of the suitable biomass prediction models that use machine learning algorithms to interpret different image parameters, (4) determination of growth rates based on a smoothed biomass curve for each genotype at the early growth stages under imposed stress conditions, and (5) estimation of the productive use of water by plants with the WUI derived from the predicted biomass. The protocol can help in phenotyping mungbean genotypes for their capacity to cover the ground rapidly in order to prevent soil moisture loss, and thus contributing to an enhancement in the canopy level water-use efficiency. Alternatively, genotypes with high WUI can serve as promising donors for improving water-use efficiency in mungbean that is to be grown in soil with poor water holding capacity.

## Conclusion

In this study, we focused mainly on optimizing the phenomics method for identifying water-use efficient genotypes (use less water to produce relatively more biomass) of a legume crop such as mungbean by applying high-throughput phenotyping approach. The model proposed by us to predict the biomass that is essential for assessing the plant growth rates, employs different parameters extracted from the images at two levels of soil moisture regimes. When integrated with the soil moisture extraction, this model enables a high-throughput non-destructive estimation of crop capacity to continue its growth even under limited available soil moisture. The method would be useful to advance our views for the accurate assessment of water-use efficiency involving a high-throughput image analysis. Water-stressed plants are a better choice than well-watered plants for assessing the genetic variation in the early stage growth rates of legume crops like mungbean. The genetic variation in a WUI at the early stages compared to the later stages of water stress would be more effective in the selection of genotypes. Surrogate traits such as WUI_AGR and WUI_RGR explain water-use efficiency at the canopy level and can help phenotyping legume crops like mungbean for tolerance to depleting soil moisture stress that occurs during drought or restricted irrigation. It can further facilitate the identification of relevant genes for the molecular marker-aided genetic improvement of the mungbean.

## Data Availability Statement

The raw data supporting the conclusions of this article will be made available by the authors, without undue reservation.

## Author Contributions

JR conceptualized, planned, and executed experiments, designed image analysis grid, analyzed data, drafted manuscript, and final editing. SR, VG, and MK contributed to the execution of experiments and drafting manuscript. PH performed mungbean experiments and generated data from phenomics platform. RG and KJ contributed to organizing data and preparation of MS draft. BH genotype selection, sharing background and seeds, generated previous field data, manuscript editing, and data interpretation and curation. RN developed genotypes and generated previous field data, interpretation, and manuscript editing. All authors contributed to the article and approved the submitted version.

## Conflict of Interest

The authors declare that the research was conducted in the absence of any commercial or financial relationships that could be construed as a potential conflict of interest.
