# Supplementary material for: Use of Phenomics for Differentiation of Mungbean (Vigna radiata L. Wilczek) Genotypes Varying in Growth Rates Per Unit of Water
Source: Front Plant Sci. 2021 Jun 21;12:692564. doi: 10.3389/fpls.2021.692564 (PMC8256871; doi:10.3389/fpls.2021.692564)
Supplement: Supplementary file 1 [file Data_Sheet_1.docx]

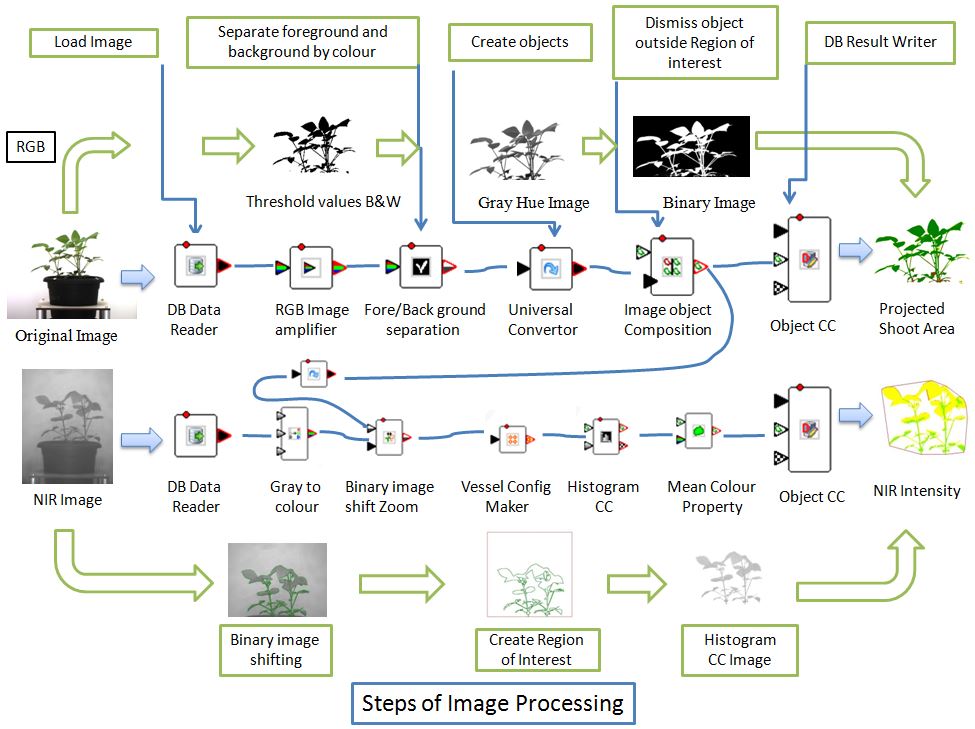


Supplementary FIGURE 1.Image analysis configuration using LemnaGrid package for image capture in the visible and NIR range of electromagnetic spectrum.

A
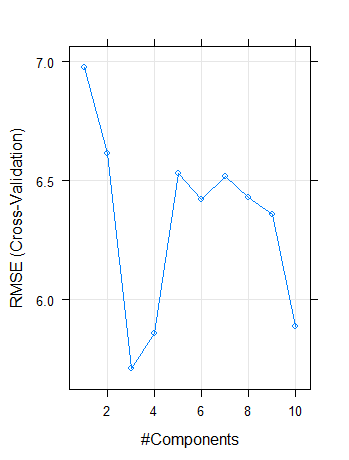
B
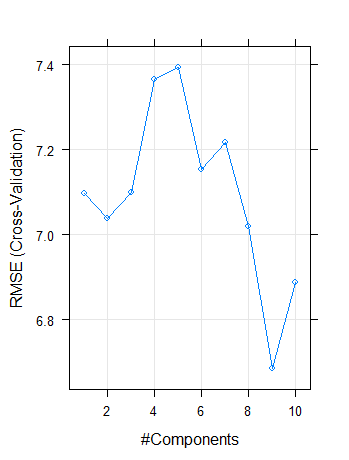


Supplementary FIGURE 2.Cross validation of (A) PLS and (B) PC models for root mean square error. x-axis explains the number of components used in prediction of biomass. The minimum number of components to achieve the lowest possible RMSE is considered as the best model. Hence PLS was considered as the prediction model better than PC.


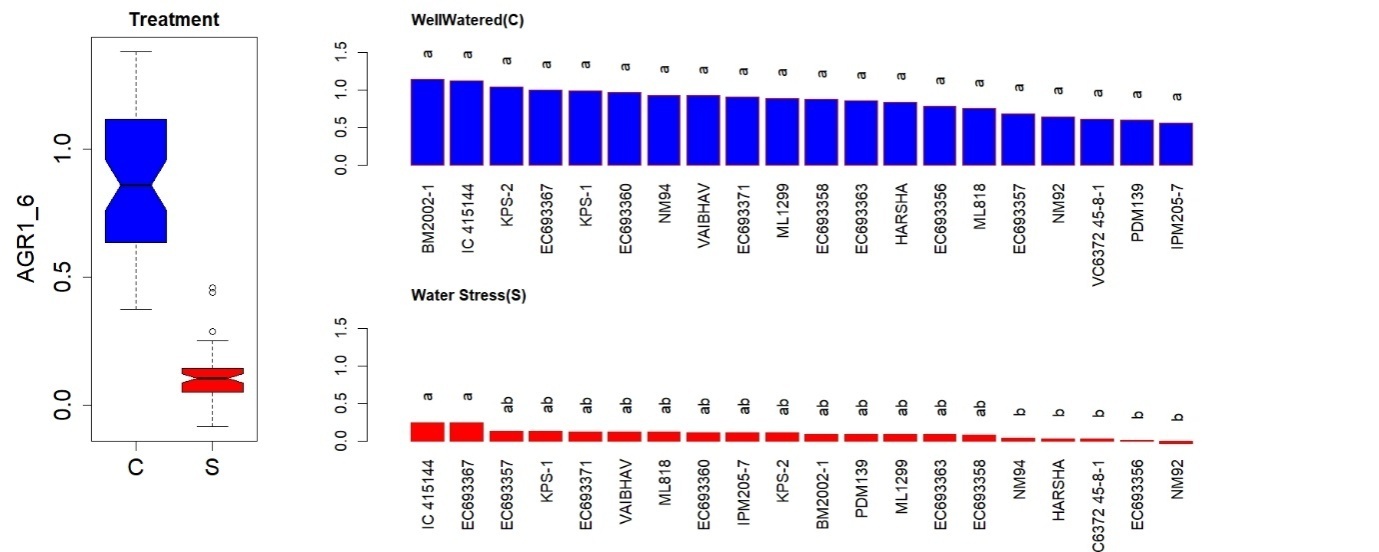


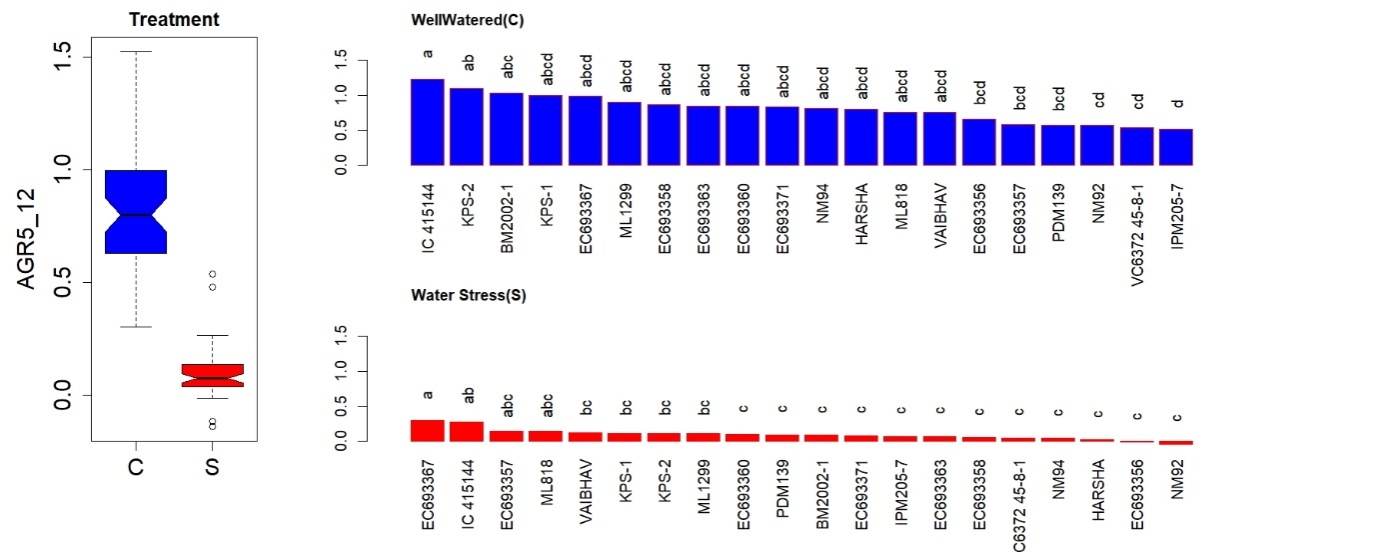


Supplementary FIGURE 3. Genetic variation in absolute growth srates (AGR) during the initial phase in response to two levels in well-watered(C) and water-stressed(S) plants; AGRs were computed for (A) 1 to 6 days and (B) 5 to 12 days. Each box in the Treatment effect is mean of 60 values(3 replications and 20 genotypes), Each bar in the genotype effect for each treatment represent mean values of 3 replications. Letters represent the significance of differences among mean values as computed by Duncan multiple range test at 0.95 CI. Genotypes with common letters are not significantly different.


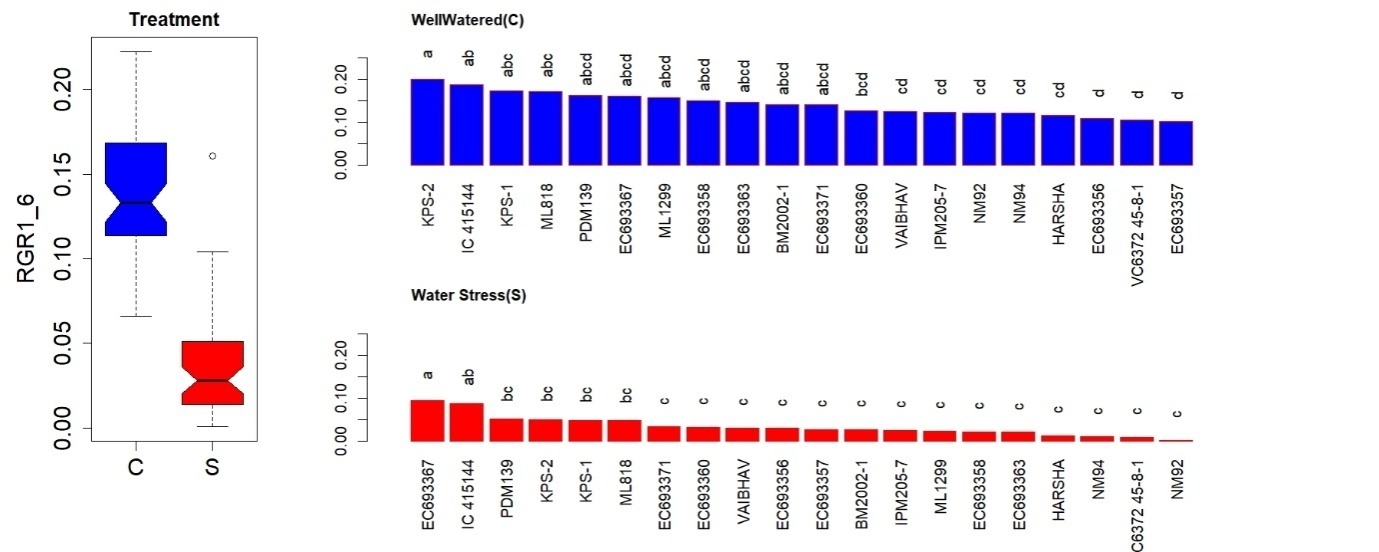


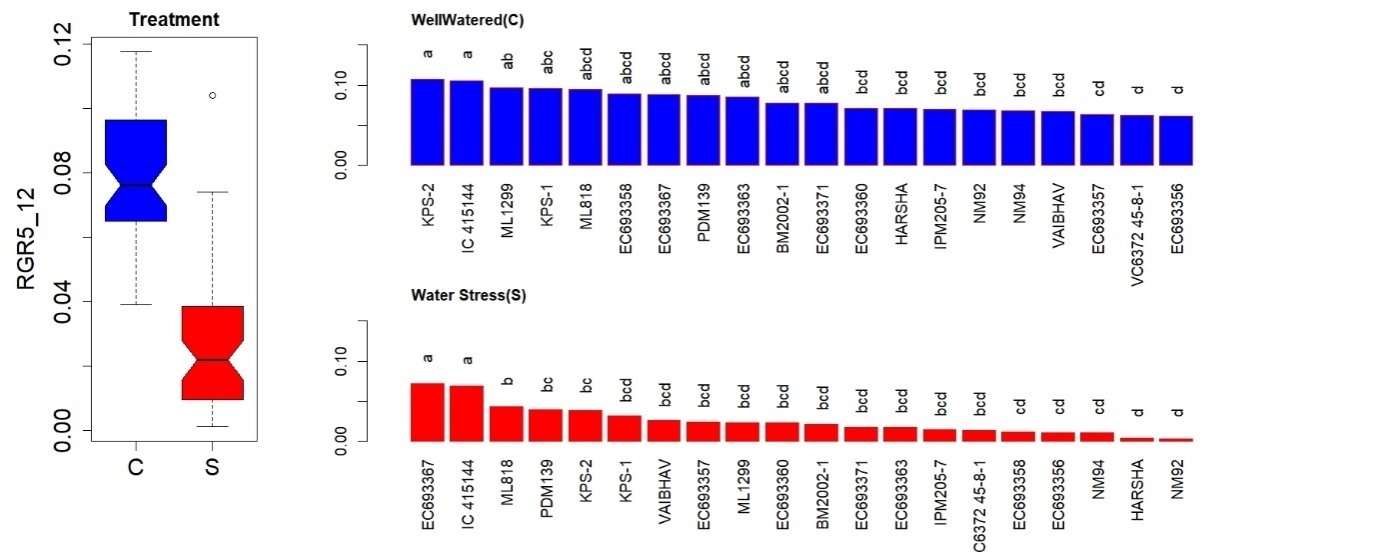


Supplementary FIGURE 4. Genetic variation in relative growth rates(RGR) during the initial phase in response to two levels in well-watered(C) and water-stressed(S) plants; RGRs were computed for (A) 1 to 6 days and (B) 5 to 12 days. Each bar represents. Each box in the Treatment effect represents 60 observations (3 replications and 20 genotypes), Each bar in the genotype effect for each treatment represent mean values of 3 replications. Letters represent the significance of differences among mean values as computed by Duncan multiple range test at 0.95 CI. Genotypes with common letters are not significantly different.
