# Supplementary material for: Use of Phenomics for Differentiation of Mungbean (Vigna radiata L. Wilczek) Genotypes Varying in Growth Rates Per Unit of Water
Source: Front Plant Sci. 2021 Jun 21;12:692564. doi: 10.3389/fpls.2021.692564 (PMC8256871; doi:10.3389/fpls.2021.692564)
Supplement: Supplementary file 2 [file Data_Sheet_2.docx]

| S.No. | Name | WorldVeg Code |
| --- | --- | --- |
| 1 | EC693356 (VC 6153B-20P) | AVMU0402 |
| 2 | EC693357 (VC 6465-8-5-2) | AVMU1202 |
| 3 | EC693358 (VC 6469-12-3-4A) | AVMU1001 |
| 4 | EC693360 (VC 6486-10-51) | AVMU1002 |
| 5 | EC 693363 (VC 6493-44-1) | AVMU1007 |
| 6 | EC 693367 (PDMA 54) | - |
| 7 | EC 693370 (TV 03717B-G) | - |
| 8 | EC 693371 (TV 01493A-G) | - |
| 9 | HARSHA | - |
| 10 | IPM 205-7 | - |
| 11 | KPS-1 (VC 1973A) | AVMU8501 |
| 12 | KPS-2 (VC 2778A) | AVMU8601 |
| 13 | ML 1299 | - |
| 14 | ML 818 | - |
| 15 | NM 92 (VC 6370-92) | AVMU9701 |
| 16 | NM 94 (VC 6371-94) | AVMU0001 |
| 17 | PAU 911 | - |
| 18 | PDM 139 | - |
| 19 | VC 3960-88 | AVMU8902 |
| 20 | VC 6372 (45-8-1) | - |
| 21 | BM 2002-1 | - |
| 22 | Vaibhav (Local check) | - |
| 23 | IC 415144 | - |
| 24 | BM 2003-2 | - |

Supplementary Table 1. Details of genotypes

Supplementary Table 2. Image parameters extracted and used for predicting fresh biomass

| **Image parameters** | |
| --- | --- |
| 1. Area | 1. Compactness |
| 1. Boundary Point Count | 1. Convex Hull Area |
| 1. Boundary Point Roundness | 1. Convex Hull Circumference |
| 1. Boundary Points To Area Ratio | 1. Excentricity |
| 1. Caliper Length | 1. Object Sum Area |
| 1. Circumference | 1. Center Of Mass X |
| 1. Center Of Mass Y | 1. Mean Color Green |
| 1. Roundness | 1. Mean Color Red |
| 1. Subobject count | 1. Mean Color Blue |

Supplementary Table 3. Relative importance of different predictors used in the PLS model for prediction of fresh biomass.

| **Predictors** | **Coefficients** | **%Contribution** |
| --- | --- | --- |
| **Negative predictors** |  |  |
| VIS_90_CIRcumference | -1.09E-04 | 65 |
| VIS_90_Roundness | -3.34E-05 | 20 |
| VIS_tv_CIRcumference | -1.96E-05 | 12 |
|  |  |  |
| **Positive predictors** |  |  |
| VIS_00_Convex.Hull.Area | 5.33E-06 | 1 |
| VIS_tv_Boundary.Point.Roundness | 6.14E-06 | 1 |
| VIS_00_CIRcumference | 1.48E-05 | 2 |
| VIS_tv_Area | 1.64E-05 | 2 |
| VIS_00_Roundness | 1.85E-05 | 3 |
| VIS_00_Area | 3.67E-05 | 5 |
| VIS_90_Boundary.Point.Roundness | 4.15E-05 | 6 |
| VIS_00_Boundary.Point.Roundness | 4.98E-05 | 7 |
| VIS_tv_Roundness | 5.10E-05 | 8 |
| VIS_tv_Boundary.Point.Count | 5.81E-05 | 9 |
| VIS_90_Area | 5.97E-05 | 9 |
| VIS_90_Boundary.Point.Count | 1.45E-04 | 22 |
| VIS_00_Boundary.Point.Count | 1.66E-04 | 25 |

Note: The table is derived from the relative importance of the predictors used in PLS model, which was finally selected for non-destructive estimation of fresh biomass of plants. Per cent contribution of selected predictors is computed from the negative and positive coefficients separately.

Supplementary Table 4. Broad sense heritability (H2) of different surrogated traits for plant growth rate and water use derived from predicted biomass on the basis of features of plant images from three views. Values were computed for the first two weeks of water stress as described in material and methods.

| Trait | Genotypic Variance | Phenotypic | *Standard Heritability | **Cullis Heritability | ***Piepho Heritability |
| --- | --- | --- | --- | --- | --- |
| Fresh Biomass | 8.73 | 18.94 | 0.46 | 0.46 | 0.46 |
| AGR | 0.72 | 1.17 | 0.61 | 0.61 | 0.61 |
| RGR | 100.22 | 122.27 | 0.82 | 0.82 | 0.82 |
| WUI_AGR | 1.84 | 2.47 | 0.74 | 0.74 | 0.74 |
| WUI_RGR | 0.35 | 0.59 | 0.60 | 0.60 | 0.60 |

*Standard broad-sense heritability method is by far the most commonly used in the plant breeding community. This method provides the advantage of a straightforward calculation along with an intuitive parameter selection. Disadvantages include a tendency to overestimate values when data is unbalanced (different number of reps per genotype in single or multi-environment setting), as it assumes balanced datasets.

**Cullis et al. (2006) propose a modern method that is widely used to account for the unbalanced scenario that plant breeder’s face in the single and multi-environment context with the advantage of not requiring every entry to have one rep. In this case, the genetic term is fitted as a random effect (BLUP). It uses the square of the standard error of the genetic estimates (across environments) to attain an approximation of the non-genetic variation.

***Piepho and Möhring(2007) developed alternative and more robust method to address the regularity in which plant breeding programs face unbalanced data, for estimating heritability were developed. The idea behind these methods is to obtain the non-genetic variance from the squared standard errors of genetic estimates, rather than attempting to deduce error variance from the plot error variance (divided by a factor that is a function of years, locations and replicates). The advantage of this method is it accounts well for unbalanced data since, as the standard errors of the genetic estimates vary in size according to replication level, so the unbalanced data is directly account for. The disadvantage is that it requires at least two measurements of each individual to obtain a standard error, because the method is based on BLUEs (fixed effects).
